# Molecular Evolution and Expansion Analysis of the NAC Transcription Factor in *Zea mays*

**DOI:** 10.1371/journal.pone.0111837

**Published:** 2014-11-04

**Authors:** Kai Fan, Ming Wang, Ying Miao, Mi Ni, Noreen Bibi, Shuna Yuan, Feng Li, Xuede Wang

**Affiliations:** 1 Institute of Crop Science, College of Agriculture and Biotechnology, Zhejiang University, Hangzhou, P.R. China; 2 Center for Molecular Cell and Systems Biology, College of Life Science, Fujian Agriculture and Forestry University, Fuzhou, P.R. China; University of Western Sydney, Australia

## Abstract

NAC (*NAM*, *ATAF1*, *2* and *CUC2*) family is a plant-specific transcription factor and it controls various plant developmental processes. In the current study, 124 NAC members were identified in *Zea mays* and were phylogenetically clustered into 13 distinct subfamilies. The whole genome duplication (WGD), especially an additional WGD event, may lead to expanding ZmNAC members. Different subfamily has different expansion rate, and NAC subfamily preference was found during the expansion in maize. Moreover, the duplication events might occur after the divergence of the lineages of *Z. mays* and *S. italica*, and segmental duplication seemed to be the dominant pattern for the gene duplication in maize. Furthermore, the expansion of ZmNAC members may be also related to gain and loss of introns. Besides, the restriction of functional divergence was discovered after most of the gene duplication events. These results could provide novel insights into molecular evolution and expansion analysis of NAC family in maize, and advance the NAC researches in other plants, especially polyploid plants.

## Introduction

Transcription factors (TFs) are a group of regulatory proteins which can regulate the expression of target genes through binding to specific *cis*-acting elements in the promoters of target genes [1], [2]. Although the genes encoding the transcription factors just accounts for a little portion in the whole genome, transcription factors are important in the regulated networks [3]. Recently it has been reported that numerous transcription factors can control many critical biological processes during plant development and growth, such as TCP, WRKY, MYB, AT-hook and E2F [4], [5], [6].

The NAC (*NAM*, *ATAF1*, *2* and *CUC2*) family is one of the largest families of plant-specific transcription factors and exists widely in various kinds of plants [7], [8]. The NAC gene was firstly reported to be related to forming shoot apical meristem and primordium in *Petunia hybia* [9], and then more and more NAC members have been investigated in a wide range of plants. Nowadays more than 100 NAC TFs have been isolated in *Arabidopsis thaliana* and *Oryza sativa* [10], [11]. Besides, the NAC family has also been found in other plant species such as *Glycine max* [12], *Solanum tuberosum* [13], *Musa acuminata* [14], *Citrus* [15], *Setaria italica* [16], *Malus domestica* [17] and *Vitis vinifera* [18]. The NAC proteins are characterized by a highly conserved N-terminal region (NAC domain) with a relatively highly divergent C-terminus region (TAR region: Transcriptional Activation Region) [11], [19]. The NAC domain (nearly 160 amino acid residues) can be further divided into five subdomains labeled from A to E and it is involved in DNA binding [20], while the TAR region usually is related to the regulation diversity and it may determine some specific functions [21]. The NAC family has been found to be involved in regulating plant growth and development, such as flower development [22], lateral root formation [23], embryogenesis [24], leaf senescence [25], secondary wall thickening [26], cell metabolism [27], seed development [28] and hormone signaling [29]. Moreover, NAC family also plays pivotal roles in response to many biotic and abiotic stresses including fungal infection [30], pathogen disease [31], salt [32], temperature [33], drought [34] and osmotic [35]. Additionally, NAC transcription factors are related to the crop yield and quality [36], [37].

As a major cereal crop in the world, *Zea mays* is not only a primary food resource, but also an important crude material. With maize genome sequencing completed, an excellent opportunity is coming to conduct whole-genome annotation, evolution and comparative genomic study in maize [38]. Moreover, previous lines of evidence have already demonstrated that NAC family may be an excellent candidate to regulate the plant development and growth, without the exception for maize. ZmNAC41 and ZmNAC100 were reported to be related to the maize defense network [39], and ZmSNAC1 can enhance tolerance to dehydration in transgenic Arabidopsis [40]. Meanwhile, ZmSWNs (NAC members) can regulate the secondary wall thickening in maize [41]. However, the related researches about the NAC family are limited in maize. Besides, duplication event has been extensively existed in many plants, but the related reports, especially in a specific family, are lacked in maize. Thus, a systematic molecular evolution and expansion analysis of the ZmNAC members is urgently required to comprehensively understand the genetics, evolution, basic function and expansion history of the NAC family in maize. In the present study, we identified NAC family in maize; and we conducted a relatively detailed research about the phylogenetics, genomic-dynamic, chromosomal localization, expansion history and expression analysis to evaluate molecular evolution and expansion history of ZmNAC family.

## Materials and Methods

### Isolation and structural analysis of the ZmNAC proteins

All NAC proteins of Arabidopsis were collected from TAIR (http://www.arabidopsis.org/index.jsp). The genome sequences of *Z. mays* were downloaded from the Phytozome database (http://www.phytozome.net/). Through searching and alignment, only the sequences containing at least four out of five conserved NAC subdomains (from A to E) were considered and used for phylogenetic analysis [15], [42]. Meanwhile, the SMART and Pfam tool confirmed that all ZmNACs contained the conserved NAM domain (PF02365) (SMART: http://smart.embl-heidelberg.de/; Pfam: http://pfam.xfam.org/). Then the genomic schematic diagram of ZmNACs was visualized using GSDS tool (http://gsds.cbi.pku.edu.cn/). Protein primary and secondary structure were calculated by ProtParam (http://web.expasy.org/protparam/) and SOPMA (http://npsa-pbil.ibcp.fr/cgi-bin/npsa_automat.pl?page=npsa_sopma.html). Besides, the chromosome location information of ZmNACs was downloaded from the Phytozome database. MapInspect software (http://www.plantbreeding.wur.nl/uk/software_mapinsp ect.html) was performed to generate the chromosomal distribution image of these ZmNAC genes [43].

### Phylogenetic analysis

Firstly, the ClustalX version 2.1 was performed to align the ZmNAC members. Then the Jones, Taylor, and Thorton (JTT) model was selected as the best-fitting amino acid substitution model through the ProtTest version 2.4 [44]. Furthermore, the maximum-likelihood analysis was run with the PhyML version 3.1 based on JTT model [45]. Besides, MrBayes version 3.1.2 was used to conduct the Bayesian analysis: two independent computations kept running until the standard deviation of split frequencies was less than 0.01 [46]. All trees were visualized through the Figtree version 1.4.0.

After phylogenetic analysis, all of the ZmNAC proteins were subjected to online MEME program to investigate conserved motifs [47] (http://meme.nbcr.net/meme/). Parameters were set as follows: optimum motif width was set to ≥6 and ≤200; Maximum number of motifs was set to 20 [43]. Sequence logos of the conservative NAC domain and TAR region were generated through WebLogo [48] (http://weblogo.berkeley.edu/).

### Gene duplication and OG identification

Gene duplication was defined according to the previous report [43]. Furthermore, tandem and segmental duplication were recognized by the chromosomal locations. Besides, the Ka/Ks ratio was used to show the selection pressure for the duplicate genes [49]. According to the synonymous substitutions per year (λ) of 6.5×10^−9^ for *Z. mays*, the approximate time of the duplication events about the duplicated pair was estimated (T = Ks/2λ×10^−6^ Mya) by substituting the calculated Ks values [50].

Additionally, the orthologous genes of the duplicate ZmNACs were searched in *S. italica*, *O. sativa* and *B. distachyon*. Firstly, the NAC proteins in *S. italica*, *O. sativa* and *B. distachyon* were obtained according to similar approach of ZmNAC identification. Then, the whole set of ZmNAC, SiNAC, OsNAC and BdNAC proteins was clustered using OrthoMCL program to identify the orthologous groups (OG) [51]. Moreover, the orthologous genes were also defined: (1) the length of aligned sequence cover >50% of the longer gene; and (2) the identity of the aligned regions >50% [52].

### Public microarry-based data analysis

The expression patterns of the ZmNAC genes in various tissues and development stages were examined from Genevestigator [53] (https://www.genevestigator.com/gv/). The ZmNAC probes were adopted according to the previous standard [54]. The expression data were hierarchically clustered and gene-wise normalized through Euclidean Distance program [55]. Besides, the EST sequences of *Z. mays* were downloaded from the GenBank EST database (http://www.ncbi.nlm.nih.gov/). The EST sequences, which were less than 0.01 in E-value and more than 80% in the identity to the ZmNAC proteins, were collected for further analysis.

### Plant materials, RNA extraction and quantitative real-time PCR

The *Z. mays* cv B73 were used to construct the expression profiles of ZmNAC family. Roots, stems and leaves were collected from three-week-old seedlings, and the fresh flowers were harvested from maize plants. Then, total RNAs were isolated from the collected samples through RNAiso Plus (TakaRa), and the first-strand cDNA was synthesized from DNase-treated RNA using PrimerScript 1st Strand cDNA synthesis kit (TaKaRa). Gene-specific qRT-PCR primers were designed according to their CDSs (Table S8) and then synthesized commercially (Generay). The quantitative real-time PCR was performed in 96-well blocks with CFX96 Realtime System (BioRad) using SYBR premix Ex Taq (TakaRa). The qRT-PCR machine was set with 40 cycles and an annealing temperature of 60°C. The *ZmHMG* gene was used as an endogenous control for all the qRT-PCR analyses. The 2^−ΔΔCt^ method was used to determine relative transcription levels and the relative expression level in the root was normalized to 1. Three biological replications were performed in all reactions. The expression patterns of ZmNACs were clustered using the Cluster 3.0 software.

## Results

### Identification of the NAC family in *Z. mays*

The NAC transcription factor is one of the largest families in plant kingdom. As a model plant, the ANAC proteins from Arabidopsis have been comprehensively investigated [8]. Thus, the ANAC sequences were regarded as a query to search against the *Z. mays* genome database with the Blastp program. After Blastp search, 211 NAC-containing sequences were identified in maize. Then the putative NAC domain in their N-terminal region was confirmed by Pfam and SMART tool. Moreover, the amino acid sequences with at least four conserved NAC subdomains (from A to E) were identified for the further analysis. After this removal, 124 sequences in maize were isolated and sequentially named as *Z. mays* NAC (ZmNAC) (Table S1). The total number of NAC genes was a little greater in *Z. mays* than in *O. sativa*, *B. distachyon* or *S. italica* (Figure S1).

### Phylogenetic investigation of ZmNAC family

**Figure 1:**
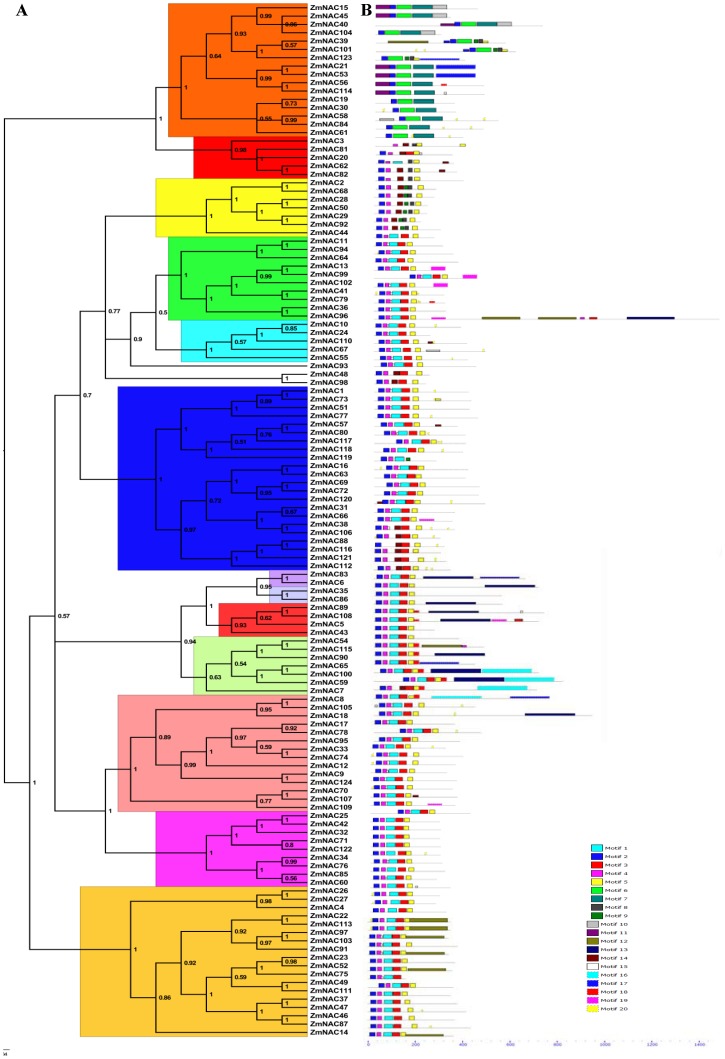
Phylogenetic relationships and putative conserved domain distributions of ZmNAC family. (A) The phylogenetic tree was generated using the Bayesian method based on the multiple alignments of ZmNAC protein sequences. The numbers in the clades are posterior probability values. The NAC subfamilies were indicated by different colors. (B) The conserved motifs were identified through MEME web server. Different motifs were represented by various colored boxes. The location of each motif can be estimated using the scale at the bottom.

**Figure 2:**
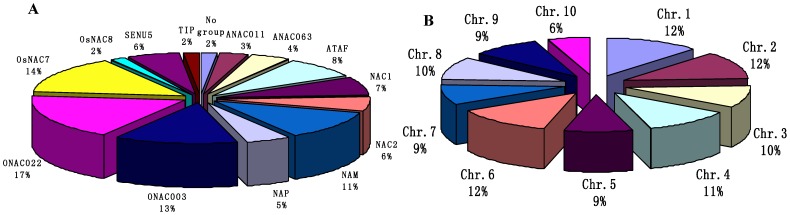
The percentage of ZmNAC proteins’ subfamily (A) and ZmNAC chromosomes localizations (B) in *Zea mays*.

The phylogenetic analysis among the identified ZmNAC and ANAC proteins was processed by the PhyML and MrBayes tool. There were similar results with high support values from each method (Figure 1A, Figure S2). According to previous studies [8], [42], the ZmNAC family was divided into 13 subfamilies through the phylogenetic analysis. The member proportion was different in each ZmNAC subfamily (Figure 2A). The ONAC022 subfamily (17%) occupied the most members, followed by OsNAC7 subfamily (14%), ONAC003 subfamily (13%) and NAM subfamily (11%). The least was OsNAC8 and TIP subfamily (2%). Besides, ZmNAC sequences were submitted to OrthoMCL clustering. With the default stringency, 37 orthologous groups (OGs) were shown and they covered all the ZmNAC members in maize (Table S2). Each subfamily contained one or more OGs, and almost the different subfamilies had distinct OGs. The OG distributions were similar to the phylogenetic classifications of the NAC family in maize.

Although all the ZmNAC proteins contain the NAC domain, their protein structures are highly diverse (Table S3). The amino acid length in the TIP subfamily, NAC2 subfamily and OsNAC8 subfamily were relatively longer, while the NAC1 and SENU5 subfamily had relatively shorter amino acid length. The similar distribution of ZmNAC family also existed in the molecular weight and protein structure.

### Conserved domain analysis in ZmNAC proteins

The MEME motif search tool was employed to identify 20 distinct conserved motifs in ZmNAC proteins (Figure 1B). According to the distribution of the 20 predicted motifs, 124 ZmNAC members can be classified into 13 distinct subfamilies, consistent with the categorization from the phylogenetic analysis (Figure 1A). The motif 2 was shared in all ZmNAC members and it corresponded to subdomain A in the NAC domain. The motif 4 or 6 is mapped to subdomain B in the ZmNAC proteins. All of the 124 ZmNACs had either motif 1, 7 or 14, which represented subdomain C. The motif 3, 7, 8 or 9 corresponding to subdomain D was also discovered in the ZmNAC family. Additionally, subdomain E matched with the motif 5 or 7 in the ZmNAC members. Besides, some subfamily-specific motifs were also found in some ZmNAC subfamilies. For example, the motif 13 was shared in TIP subfamily, and the NAC2 subfamily contained the motif 18.

All the conserved residues from subdomain A to subdomain E were shown in NAC domain through sequence alignment of NAC proteins in *Z. mays* (Figure S3). Although the TAR region was relatively divergent, some conserved subdomains existed in TAR region among some NAC subfamilies. Through WebLogo program, subdomain A, C and D showed a high conservation among the ZmNAC members, whereas subdomain B and E were relatively divergent. In addition, some conserved subdomains were also discovered in TAR region of some ZmNAC subfamilies (ATAF, NAC2, NAM, ONAC003, ONAC022 and OsNAC7 subfamily). But none of the 6 conserved subdomains corresponded to any known domain. The DNA binding domain (DBD) was highly conserved in the subdomain C, and a degenerate bipartite nuclear localization signal (NLS) was also detected in the subdomain D.

### Intron dynamic detection of ZmNAC family

Gene structure and intron phase were investigated among the ZmNAC family. According to the number of exons/introns, the ZmNAC family could be divided into five types (Table S3). The first type contained 67 ZmNACs and had three exons. The second type had no intron as 6 ZmNAC belonged to this type. 19 genes formed the third type with only one intron. The fourth type had 24 genes which contained three, four or five introns. The fifth type mainly covered genes with the most exons (the number is 7, 8, 9 or 14). The largest number of the exons was found in ZmNAC39 with 14 exons. The first type of gene structure included most of ZmNAC subfamily, while the ANAC063 subfamily covered the second type. The third type appeared in the 7 subfamilies, and 8 subfamilies had the fourth type of gene structure.

**Figure 3:**
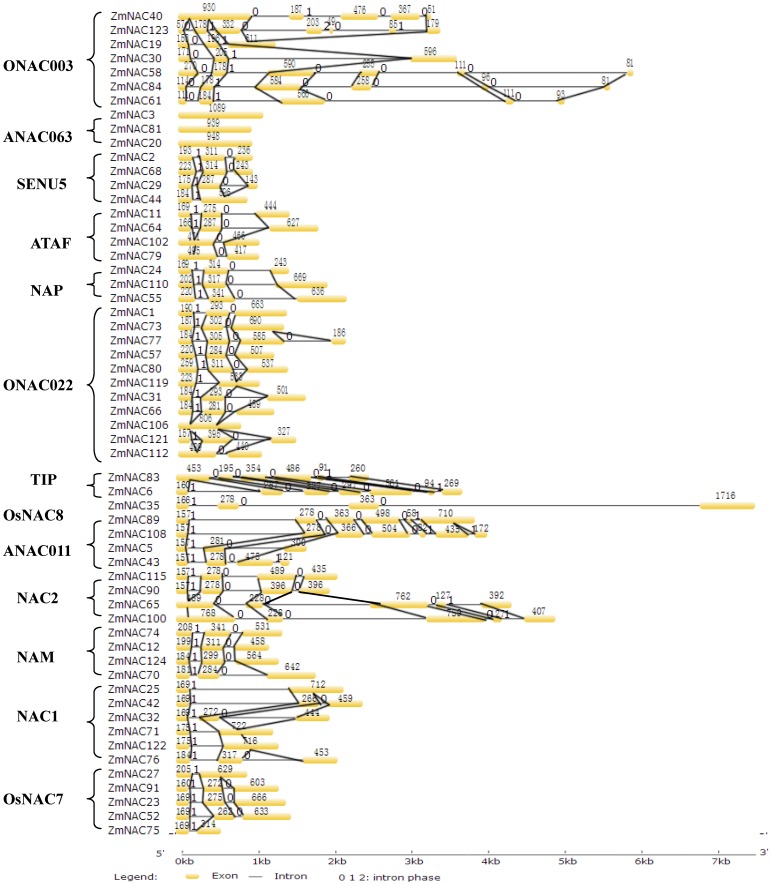
Gene structure dynamics of some ZmNAC members. Gene structures of ZmNAC were performed by the GSDS software. Subgroup designations are indicated by brackets.

Intron position and phase were examined to unravel evolutionary process of the ZmNAC gene structure (Figure 3). The primary gene structure contained three exons and two introns in ZmNAC family. The first (140 bp–230 bp) and second exon (230 bp–340 bp) showed the relatively conserved exon length. However, the divergent length and number mainly existed after the second exon, especially for ONAC003, TIP, ANAC011 and NAC2 subfamily. Besides, the gain and loss of introns resulted in the different gene structures. Some SENU5, NAC1 and OsNAC7 members lost an intron in the second and third exon, while the similar loss in the first and second exon mainly existed in the ATAF, TIP and NAC2 subfamily. Moreover, the ONAC022 and NAM subfamily contained the above-mentioned two intron loss. In addition, the intron gain usually existed in the third exon, especially for the ONAC003, TIP, ANAC011 and NAC2 subfamily. In contrast, the NAP subfamily did not undergo any changes in the gene structure, and still had three exons. Furthermore, there were not any introns in the ANAC063 members. Meanwhile, the first and second intron phase is 1 and 0 in most ZmNAC subfamilies. However, the ONAC003 subfamily had totally different intron phase, that is 0 and 1 in the first and second intron phase; and no intron phase were found in the ANAC063 subfamily.

### Genomic locations and duplication of ZmNAC genes

**Figure 4:**
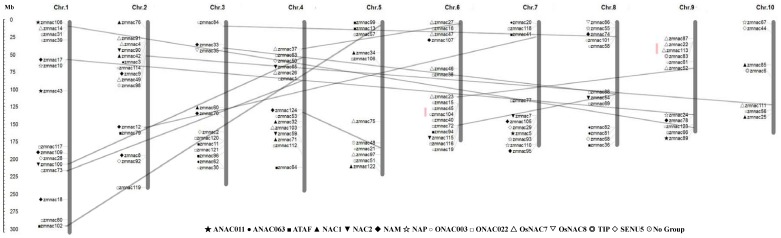
Chromosomal locations of ZmNAC genes on all 10 chromosomes. The scale is megabases (Mb). Markers before the gene names indicate the NAC subfamily. The red lines mark the tandem duplication of the ZmNAC genes, and genes related to segmental duplication are joined by gray lines.

A total of 124 ZmNAC members were mapped into 10 chromosomes in maize (Figure 4; Table S4). Every one of the 10 chromosomes contained the ZmNAC genes. The 124 ZmNAC genes distributed unevenly across the *Z. mays* chromosomes (Figure 1B). Chromosome 6 had the largest number of ZmNAC genes with 16 members, followed by chromosome 1 and chromosome 2 with 15 genes each. In contrast, only 7 members were located on chromosome 10. Besides, several ZmNAC genes clustered within a short distance such as the top of chromosome 2 and the bottom of chromosome 6.

**Figure 5:**
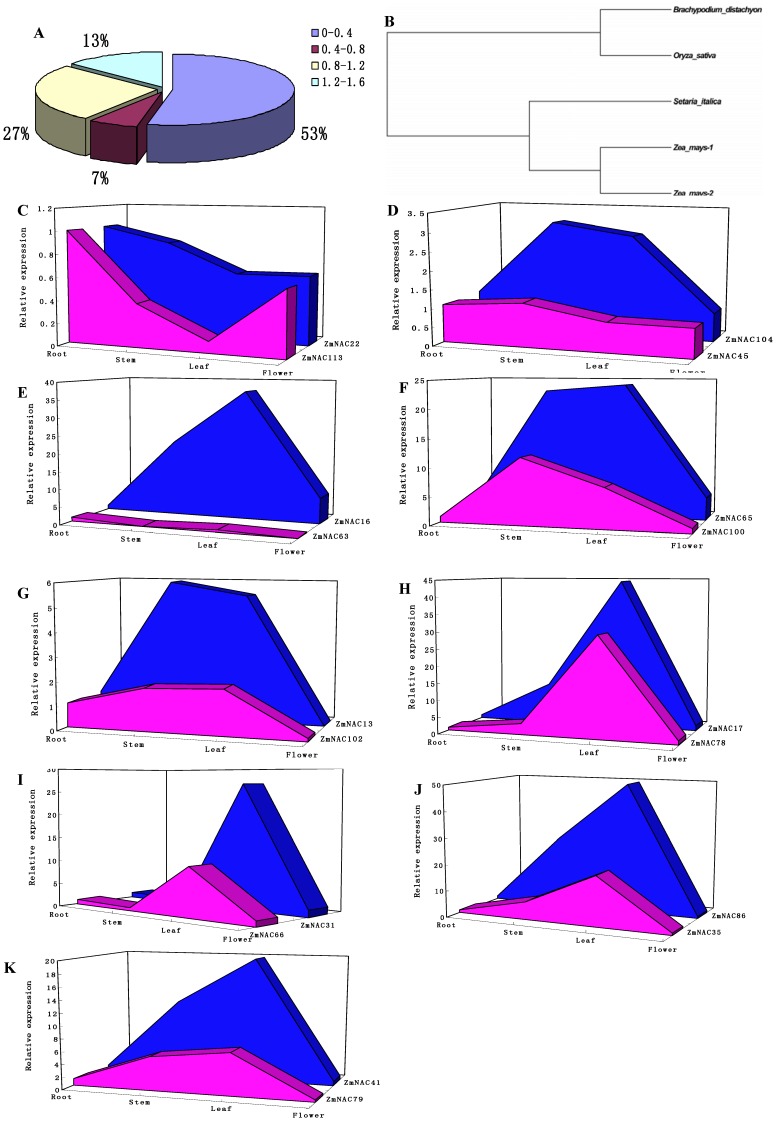
Age distribution, evolutionary history and expression analysis of the duplicated ZmNAC genes. (A) Age distribution of the duplicated ZmNAC genes based on Ks values. (B) Phylogenetic relationships among the duplicated ZmNACs and their orthologous genes in other three monocots. (C–K) Expression pattern of some duplicated ZmNAC genes. The expression values of duplicated genes obtained from quantitative real-time PCR were compared in different tissues.

15 gene duplication events were identified in maize (Figure 4, Table 1). Among them, 4 duplication events occurred in the ONAC022 subfamily, and OsNAC7 and ONAC003 subfamily had 3 duplication ones. Based on the sequence analysis and the chromosomal distribution, 13 gene pairs were identified to be involved in the segmental duplication events, while other 2 pairs were related to the tandem duplication events. Meanwhile, the expression patterns of some duplicated genes were also analyzed in the different tissues. Out of the 15 pairs of duplicated genes, 9 pairs were selected to reveal the expression relationship. The average signal values for the 9 pairs of duplicated genes were shown as an area-diagram. In the tandem duplication events, one pair of genes (ZmNAC22/113) had highly similar expression level, whereas another one pair (ZmNAC45/104) showed the divergent expression pattern (Figure 5C, Figure 5D). In the segmental duplication events, two pairs of genes (ZmNAC16/63 and ZmNAC65/100) had different expression profiles in the tissue tested (Figure 5E, Figure 5F). In each of other 8 pairs, their member share very similar expression model in different tissues, although the amplitude of expression is a little different in paired partners (Figure 5G–Figure 5K). Besides, the Ka/Ks ratio of each duplicated gene pairs was calculated to estimate the molecular evolutionary rates (Table 1). The Ka/Ks ratios from 12 duplicated gene pairs were less than 1, while in other 3 duplicated gene pairs are more than 1. Moreover, Ks age distribution of ZmNAC paralogs was constructed to predict the burst of duplication (Figure 5A). Most of Ks value were less than 0.4, and their corresponding duplication age might have been less than 30 million years ago (Mya). Furthermore, there are 4 duplicated pairs of which the Ks values are between 0.9 and 1.1, and their duplications might occur in 70–87 Mya. Additionally, the Ks values were less than 0.06 in the tandem duplicated pairs, and its corresponding events might occur in less than 0.5 Mya.

**Table 1:** Ka/Ks analysis and estimated divergence time for the ZmNAP duplicated genes.

Duplicated gene 1	Duplicated gene 2	Subfamily	Ka	Ks	Ka/Ks	Purifying selection	Age(MYA)
ZmNAC1	ZmNAC73	ONAC022	0.075446	0.383504	0.196728	yes	29.500308
ZmNAC13	ZmNAC102	ATAF	0.927048	1.57896	0.587125	yes	121.45846
ZmNAC16	ZmNAC63	ONAC022	1.00354	0.987456	1.01629	no	75.958154
ZmNAC17	ZmNAC78	NAM	0.043229	0.292898	0.14759	yes	22.530615
ZmNAC21	ZmNAC53	ONAC003	0.054503	0.195477	0.278822	yes	15.036692
ZmNAC22	ZmNAC113	OsNAC7	1.73E-07	1.10E-06	0.157044	yes	8.48E-05
ZmNAC23	ZmNAC52	OsNAC7	0.97065	1.12054	0.866235	yes	86.195385
ZmNAC31	ZmNAC66	ONAC022	0.956598	1.24751	0.766806	yes	95.962308
ZmNAC35	ZmNAC86	OsNAC8	0.087589	0.217746	0.402251	yes	16.749692
ZmNAC41	ZmNAC79	ATAF	0.037907	0.603107	0.062852	yes	46.392846
ZmNAC45	ZmNAC104	ONAC003	0.013719	0.005979	2.29461	no	0.4599123
ZmNAC49	ZmNAC111	OsNAC7	0.019899	0.33814	0.058847	yes	26.010769
ZmNAC58	ZmNAC84	ONAC003	0.993472	1.02646	0.967863	yes	78.958462
ZmNAC65	ZmNAC100	NAC2	1.01946	0.937213	1.08776	no	72.093308
ZmNAC69	ZmNAC72	ONAC022	0.085138	0.221639	0.384129	yes	17.049154

Furthermore, orthologous genes of some 15 duplicated gene pairs were detected in *S. italica*, *O. sativa* and *B. distachyon* (Table S5). For most of these duplicated genes, their corresponding orthologous genes can be discovered in three monocots. Then phylogenetic tree including these paralogous and orthologous genes was employed to estimate the relative time of the duplication events (Figure S4). Phylogenetic analysis indicated that the dominant topology between two *Z. mays* paralogs and the *S. italica* ortholog is ((*Z. mays*, *Z. mays*) *S. italica*) (Figure S4A–N, Figure 5B). But a different topology existed in one duplication event (ZmNAC 49/111), which might occur before the divergence between the *Z. mays* and *S. italica* (Figure S4O).

### Expression profiles of ZmNAC members

The *in silico* frequencies of ZmNAC ESTs provide information to the basic statistical analysis of gene expression profiles in different tissues (Figure S5, Table S6). The *Z. mays* ESTs sequenced at Stanford Universtiy mainly contained three large cDNA libraries, which covered root (10611 ESTs), immature ear (9277 ESTs) and leaf (5871 ESTs). Through EST screening, 15 ZmNACs were found to express in the three libraries. 7 ZmNAC genes were expressed in immature ear and 4 members of these genes belonged to NAM subfamily. 3 NAC2 members were preferentially expressed in the root, and shoot EST library contained 2 ZmNAC genes from the ONAC022 subfamily.

**Figure 6:**
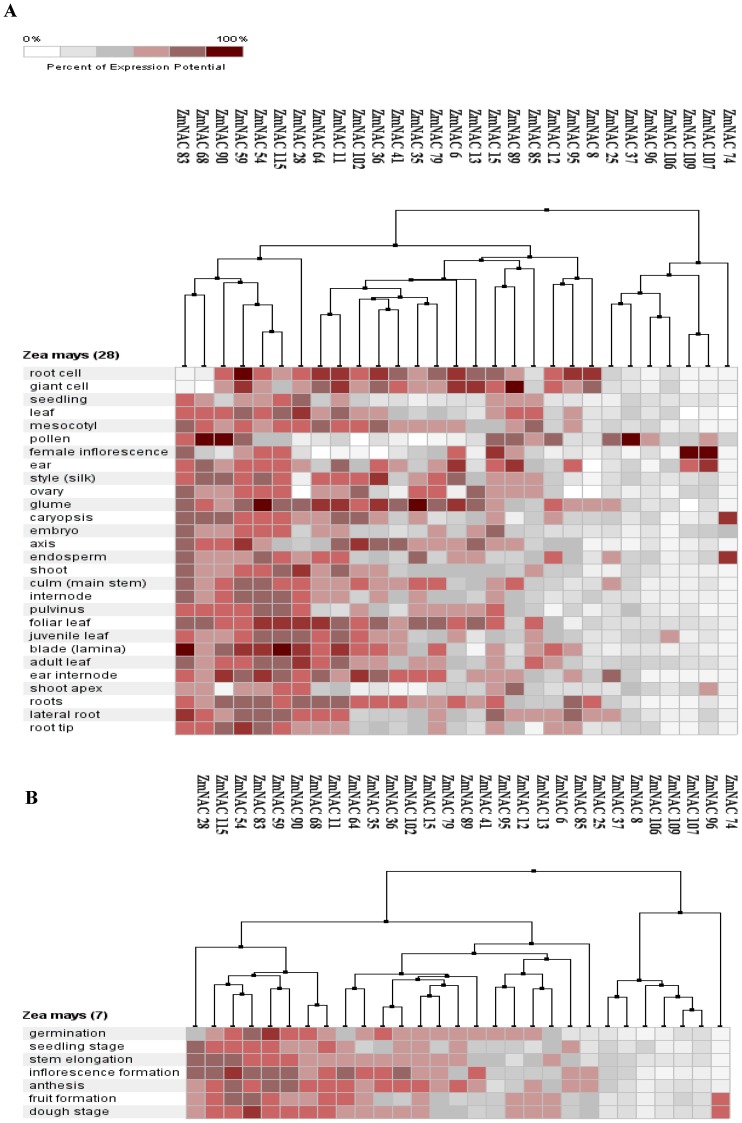
Hierarchical clustering of tissue-specific (A) and developmental (B) expression patterns of some ZmNAC genes by Genevestigator database. Color bar at top indicates percent of expression potential. The hierarchical clustering was calculated by Euclidean distance.

Many microarray chips of maize were available on the Genevestigator database. 29 ZmNAC genes have their corresponding probe sets in the ZM-15K dataset (Table S7). First, the tissue-specific expression pattern was performed to show spatial feature of these ZmNACs (Figure 6A). The NAC2 subfamily (ZmNAC 115, ZmNAC 54, ZmNAC 59 and ZmNAC 90) and SENU5 subfamily (ZmNAC 28 and ZmNAC 68) were extensively expressed in almost of all the tested tissues. The expression level of the NAC2 subfamily was very high in the root cell, lateral root and root tip, while the SENU5 subfamily expressed abundantly in the leaf and shoot. Moreover, most of the ATAF subfamily (ZmNAC 13, ZmNAC 79, ZmNAC 41, ZmNAC 36, ZmNAC 102, ZmNAC 11 and ZmNAC 64) had relatively high transcription levels in root cell, glant cell, glume and foliar leaf. Furthermore, the expression of NAM subfamily (ZmNAC74, ZmNAC107, ZmNAC109, ZmNAC8, ZmNAC95 and ZmNAC12) was only restricted to very few tissues that were mainly involved in the flower development. Meanwhile, the ZmNAC expression was also investigated under 7 developmental stages in maize (Figure 6B). Most of the NAM members were found in low transcription level over all stages, while relatively high expression level was detected in the NAC2 and SENU5 subfamily. The NAC1 subfamily and the ATAF subfamily had relatively high expression level in the inflorescence formation and anthesis stage. The expression level of some ATAF members was relatively high at the germination stage.

qRT-PCR analysis was performed to examine the expression pattern of 27 selected ZmNAC in root, stem, leaf and flower. As it is shown, two NAC2 members (ZmNAC65 and ZmNAC100), two OsNAC8 members (ZmNAC35 and ZmNAC86), two TIP members (ZmNAC6 and ZmNAC83) and two SENU5 members (ZmNAC29 and ZmNAC50) exhibited high expression level in the leaf and stem. On the contrary, the OsNAC7 subfamily (ZmNAC22 and ZmNAC113) had the opposite expression pattern. Furthermore, the NAM subfamily (ZmNAC17 and ZmNAC78) and one NAC1 member (ZmNAC34) showed relatively lower transcription level in root than in other three tissues. The expression profile of some ATAF members (ZmNAC13, ZmNAC41, ZmNAC79 and ZmNAC102) and the ONAC003 subfamily (ZmNAC45, ZmNAC58 and ZmNAC104) in the flower is the lowest in all the tissues tested. However, the leaf showed the relatively higher transcription level of the ONAC022 subfamily (ZmNAC16, ZmNAC31, ZmNAC63, ZmNAC66 and ZmNAC69). In addition, the ZmNAC81 (ANAC063 subfamly) and ZmNAC 67 (NAP subfamily) had the high transcription level in root, and ZmNAC 67 also showed high expression level in flower.

## Discussion

As an important monocot, the announcement of maize genome sequencing offers a good opportunity to further investigate the monocot and plant evolution in general. The present research mainly analyzed the ZmNAC molecular evolution, and its corresponding expansion patterns in monocot.

### The identification and function of ZmNAC family

In the present study, 124 NAC members were isolated in *Z. mays* using Arabidopsis NAC proteins as query (Table S1). This number, however, can be a conservative estimate for ZmNACs in maize, because the selected ZmNACs contained at least four conserved NAC subdomains (A to E) [15]. According to previous studies [8], 13 NAC subfamilies were phylogenetically clustered in the ZmNAC family (Figure 1A, Figure S2). The ONAC022, OsNAC7, ONAC003 and NAM subfamily had the most members, while other subfamilies contained relatively few ZmNACs (Figure 2A). The similar distribution of NAC family also can be found in banana [14], chinese cabbage [57] and so on. Then the protein structure, orthologous group, gene structure and conserved motifs confirmed the similar classification of NAC family in maize (Figure 1B, Figure 3, Table S2 and Table S3). In addition, NAC family plays significant roles in plant-specific processes [7], [19], [21], [58]. The microarray chips were used to investigate ZmNAC’s functions in the developmental stages and tissues (Figure 6). However, only 29 ZmNACs have their corresponding probes in the Genevestigator database (Table S7). These arrays from ESTs, not whole genome coverage, maybe partially lead to the relatively low coverage of ZmNAC members. Similar phenomenon also existed in the soybean [54]. Furthermore, combing with the EST frequency, NAC2 subfamily was discovered to express highly in the root, which indicated that NAC2 subfamily in maize may be related to root development. But two NAC2 members (ZmNAC65 and ZmNAC100) showed high expression level in the leaf and stem by qRT-PCR analysis. The different result maybe partially due to the extensively expression of NAC2 subfamily in almost of all the tissues and all the developmental stages. Meanwhile, the NAM subfamily in maize may be involved in the flower development, which has been confirmed in the NAM subfamily of Arabidopsis and petunia [9], [59]. Moreover, ZmNAC102, one of ATAF members, has been previously found to control the lateral root development through miRNA164-directed cleavage [56], and due to relatively high expression in root, other ATAF members might have similar function in maize like ZmNAC102. Thus, the functions within the NAC subfamily were relatively conserved throughout maize and plant kingdom; and different subfamilies might have different biological functions, mainly because of distinct structure, especially in the TAR region. Additionally, it is suggested that the NAC family is a perfect candidate to regulate the plant growth and development.

### Expansion of the NAC family in *Z. mays*

**Figure 7:**
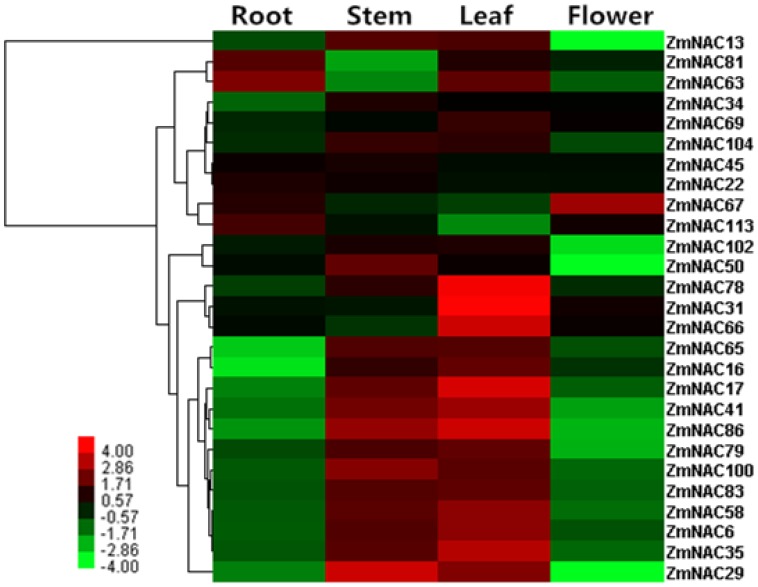
Heat map representation and hierarchical clustering of ZmNAC genes in *Zea mays* across different tissues. The color bar represents the relative signal intensity value.

*Z. mays* is an important model plant for fundamental research on evolutionary history. In the current research, we isolated 124 NAC transcription factors in maize (Table S1). Then the NAC members were also identified in *O. sativa*, *B. distachyon* and *S. italica* through the same method (Figure S1). The number of NAC members revealed that the NAC family was a little larger in maize than in other three monocots, and it may be due to the larger genome. Meanwhile, the expansion of NAC members in maize confirmed some previous observations which the same subfamily from different plants shared the similar functions (Figure 6, Figure7). Through sequence and OG analysis, the duplicated genes of NAC family were discovered in maize (Table 1, Table S2). According to the chromosomal distributions, most of the duplicated genes located in different chromosomes (Figure 4, Table S4). Meanwhile, the duplication events mainly concentrated on some specific subfamilies including the ONAC022, NAC2, ATAF, NAM, ONAC003, OsNAC8 and OsNAC7 subfamily, whereas no duplicated ones were detected in other subfamilies. Thus, it revealed the strong expansion preference for some NAC subfamilies. As an important paleopolyploid plant, maize has experienced a meaningful process from an ancient allotetraploid to a genetically diploid state [38]. During this process, numerous chromosomal breakages and fusions resulted in gene losses and gene retentions as duplicate orthologs. Based on the analysis of NAC family in maize, retention of ZmNAC members as duplicates is not random, e.g. the expansion preference of some ZmNAC subfamilies. Thus, it is indicated that the ZmNAC subfamily might have various retention rate during the plant evolution and the ZmNAC reference contributes to basic researches about plant evolution, especially polyploid plants e.g. *Gossypium hissutum*, *Brassica campestris* and *Triticum aestivum*. In addition, the gene losses and retentions might be also associated to the related functions during the plant evolution. In addition, NAC family can control a variety of plant growth and development processes [7], [8]. Thus, according to the various retention rate of the ZmNAC subfamily, some putative key NAC members can be selected to regulate some phenotypes in the polyploid plants.

Previous studies revealed that the maize genome has undergone several rounds of genome duplication, including a paleopolyploid duplication event (about 70 Mya) [60] and an additional whole-genome duplication (WGD) event (about 12 Mya) [61]. In the ZmNAC family, most of duplication events (53%) might occur about less than 30 Mya, while 27% duplicated ones between 70 Mya and 87 Mya. Moreover, the othologous genes of these duplicated genes were isolated in *O. sativa*, *B. distachyon* and *S. italica* (Table S5). Through phylogenetic analysis, the dominant topology in four monocots is ((*O. sativa*, *B. distachyon*), ((*Z. mays*, *Z. mays*) *S. italica*)) (Figure 5B, Figure S4). Only one pair of duplicated genes (ZmNAC 49/111) showed the different topology (Figure S4O). The divergence may be attributed to the extensive rate variation among species [62]. Thus, it was concluded that the ZmNAC duplication events might mainly occur after the divergence of the lineages of *Z. mays* and *S. italica*. Above all, the expansion of NAC family in *Z. mays* arose from the whole genome duplication events, might mainly occur after the divergence of the lineages of *Z. mays* and *S. italica*, and the additional WGD event might have more influence on the expansion of ZmNAC members. The expansion of NAC family in other plants (e.g. *G. raimondii* and *P. trichocarpa*) has been similarly related to the whole genome duplication events [63], [64].

Compared with other monocots, maize has undergone relatively more chromosomal breakages and fusions [38]. Meanwhile, the tandem duplication events in ZmNAC family might occur in less than 0.5 Mya (Table 1). Therefore, after an additional WGD event, interchromosomal rearrangements also have a certain effect on the expansion of ZmNAC members during the return to a genetically diploid state (Figure 4). Coincidently, this finding is consistent with the expansion of other gene families including ERF family and F-box family [65], [66].

### The conserved motifs’ role during ZmNAC expansion

The NAC family has a conserved structure including a highly conserved NAC domain in the N-terminal domain and a relatively divergent C-terminal domain [7], [8]. Without exception, the ZmNAC family also contained the NAC domain and TAR region (Figure 1B, Figure S3). Then through sequence alignment, we found, subdomain A, C and D were tightly conserved, while subdomain B and E were relatively divergent. The similar phenomena also exist in NAC family of other plants [8]. In addition, subdomain C was found to have a DNA binding domain (DBD), which indicated that subdomain C may be involved in the DNA binding [20]. Moreover, the DBD sequence (WKATGXD[K/R]) resembled the DBD sequence (WRKYGQK) in the WRKY family [67]. This sequence similarity revealed that the NAC family and WRKY family might share the similar evolutionary history in maize, and the ZmNAC family may originate from the protist WRKY family in WRKY-GCM1 superfamily [67]. Furthermore, the putative nuclear location signal (NLS) was found in the subdomain D, which indicated their nuclear localization. The transient expression of some NAC members from other plants also suggested that most of the NACs are a nuclear protein [25], [42]. Thus, the NAC domain played an important role in some conserved functions such as stress responses.

Although the TAR region was divergent in the ZmNAC family, the TAR region within the same subfamily was relatively conserved, especially for the ATAF, NAC2, NAM, ONAC003, ONAC022 and OsNAC7 subfamily (Figure 1, Figure S3). This also may be the reason why the same NAC subfamily might regulate similar processes in different plants. Other plant-specific transcription factors have similar conditions, such as WRKY and MADS family [68], [69].

### Gene structure dynamic during ZmNAC expansion

Intron is a characteristic feature of the eukaryotic genes, and the relatively little selective pressure acts on intron [70]. Most of ZmNAC had three exons and two introns (Figure 3, Table S3). The first and second exon was relatively conserved in length, partially due to encoding the conserved NAC domain. However, there were different length and intron insertions in the third exon, especially for ONAC003, TIP, ANAC011 and NAC2 subfamily. The divergence of gene structure might mainly come from the exonization of intronic sequences or pseudoexonization of exonic sequences. Furthermore, compared with NAC family in other plants, the ZmNAC genes contained more large introns, partially because of insertion of repetitive elements [38]. Meanwhile, gene structure also confirmed the previous classification of ZmNAC subfamily (Figure 1A). Besides, the first and second intron phase were similar in ZmNAC subfamilies except the ONAC003 and ANAC063 subfamily. The ANAC063 subfamily did not have any intron phases, while first and second intron phase in the ONAC003 subfamily was entirely different from other subfamilies. This indicated that the expansion of ANAC063 and ONAC003 subfamily might be relatively independent from other subfamilies in maize. Above all, gain and loss of introns might take important parts in the expansion of ZmNAC members, and ANAC063 and ONAC003 subfamily perhaps have experienced the different evolutionary history from other ZmNAC subfamilies.

### Functionalization of the ZmNAC duplicated genes during the maize evolution

During the evolutionary process, duplication genes might have experienced functionalization at the level of gene expression to retain in the genome [71]. In this study, 15 pairs of duplicated genes were identified in the ZmNAC family (Table 1). Most of Ka/Ks ratio was less than 1 in these duplicated gene pairs, indicating that the ZmNAC genes have mainly undergone purifying selection pressure with limited functional divergence after the duplications. Then, the expression patterns of six pairs of duplicated genes (Ka/Ks <1) were very similar in the different tissues through qRT-PCR analysis (Figure 5C, Figure 5G–K). These results indicated that most of the duplicated genes of ZmNAC family might retain some essential functions during sequent evolution. The retaining similar expression profiles may be related to highly similar amino acid sequence of these duplicated genes. Consistent with this observation, two duplicated ZmNAC paralogs (ZmNAC 36/96) were induced in response to *C. graminicola* infection [39]. However, this reported paralogs was not regarded as duplicated genes in our study, mainly because of the relatively low sequence similarity. Besides, there was three pairs of duplicated gene with a Ka/Ks ratio more than 1, which suggested strongly accelerated evolution with positive selection. Then three pairs of the duplicated gene showed the divergent expression pattern (Figure 5D, Figure 5E, Figure 5F). This result indicated that this pair of duplicated genes might have undergone significant diversification after duplication. Above all, functional divergence was limited in ZmNAC family after most of the gene duplication events.

In summary, 124 NAC transcription factors were identified in *Z. mays* in this study. Moreover, maize contained much more NAC members than other three monocots, and the duplication events (mainly segmental duplication event) might occur after the divergence of the lineages of *Z. mays* and *S. italica*. Meanwhile, the whole genome duplication, especially an additional WGD event, might take important roles during the ZmNAC expansion and this expansion has a certain NAC subfamily preference in maize. Furthermore, the expansion of ZmNAC members may be related to gain and loss of introns. Moreover, the restriction of functional divergence was concluded after most of the gene duplication events. Besides, the specific motifs and functions were highly conserved within the same subfamilies. The findings here provide the researchers a novel draft about molecular evolution and expansion history of NAC family in *Z. mays* and offer a good opportunity to further investigate NAC family in plants.

## Supporting Information

Figure S1 **A simplified phylogeny of three monocots.** The total number of NAC family is showed in each species.
(TIF)

Figure S2 **Phylogenetic tree of NAC proteins from** ***Z. mays*** **and Arabidopsis.** Amino acid sequences were aligned using ClustalW and the maximum likelihood was generated through PhyML software. Names beginning with “ZmNAC” are NAC domains in *Z. mays.* All of ANACs in *A. thaliana* were achieved from TAIR. The NAC proteins of *Z. mays* are isolated as listed in . The subfamilies within the NAC family, as designated by Ooka et al (2003), are grouped by colors.
(TIF)

Figure S3 **The conserved domain analysis in ZmNAC protein using WebLogo program.** Sequence logos of NAC domain (A) and TAR region (B) among ZmNAC family. The height of letter designating the amino acid residue at each position represents the degree of conservation. The numbers on the x-axis represent the sequence positions in its corresponding conservative domains. The y-axis represents the information content measured in bits.
(TIF)

Figure S4 **Phylogenetic relationships among the 15 pairs of duplicated ZmNAC genes and its corresponding orthologous genes in other three monocots.** The Bayesian method was used to construct the phylogenetic tree. The numbers in the clades are posterior probability values.
(TIF)

Figure S5 ***In silico*** **frequency of** ***Z. mays*** **NAC genes ESTs.** The EST frequencies of 14 ZmNAC genes were calculated through screening three EST libraries representing three different tissues.
(TIF)

Table S1 **ZmNAC proteins identified in** ***Z. mays*** **.**
(PDF)

Table S2 **List of the orthologous groups of ZmNAC proteins through OtrhoMCL clustering.**
(PDF)

Table S3 **The structural analysis of ZmNAC identified in this study.**
(PDF)

Table S4 **Genomic locations of NAC genes in** ***Z. mays*** **.**
(PDF)

Table S5 **The duplicated ZmNAC genes and its corresponding orthologous genes in other three monocots.**
(PDF)

Table S6 **EST frequencies of ZmNAC genes.**
(PDF)

Table S7 **Selected some ZmNAC genes used for coexpression analysis in Genevestigator database.**
(PDF)

Table S8 **PCR primers used in this study.**
(PDF)
