# Supplementary figures and images for: Molecular Evolution and Expansion Analysis of the NAC Transcription Factor in Zea mays
Source: PLoS One. 2014 Nov 4;9(11):e111837. doi: 10.1371/journal.pone.0111837 (PMC4219692; doi:10.1371/journal.pone.0111837)

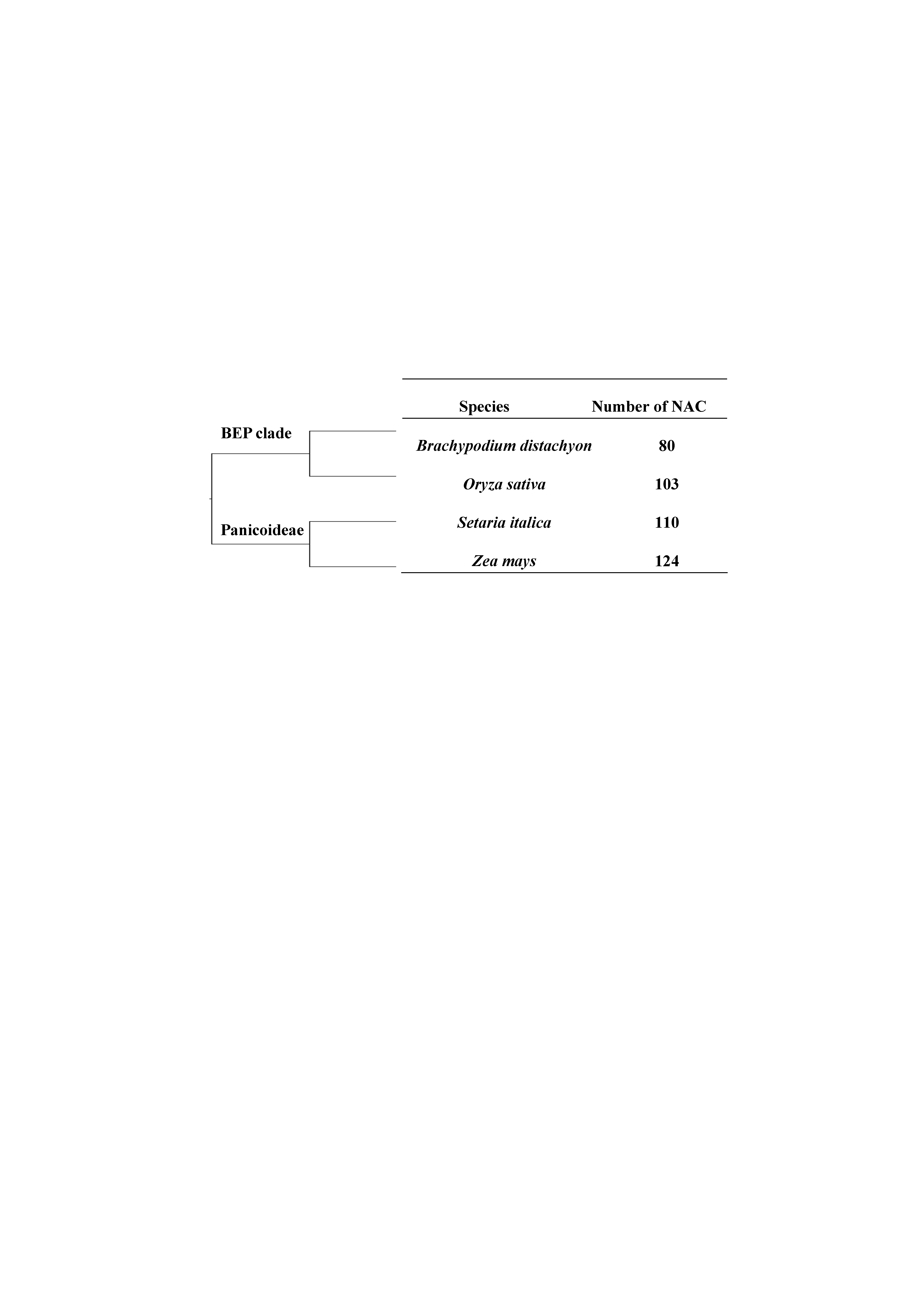

Supplement: Figure S1 — A simplified phylogeny of three monocots. The total number of NAC family is showed in each species. (TIF) [file pone.0111837.s001.tif]

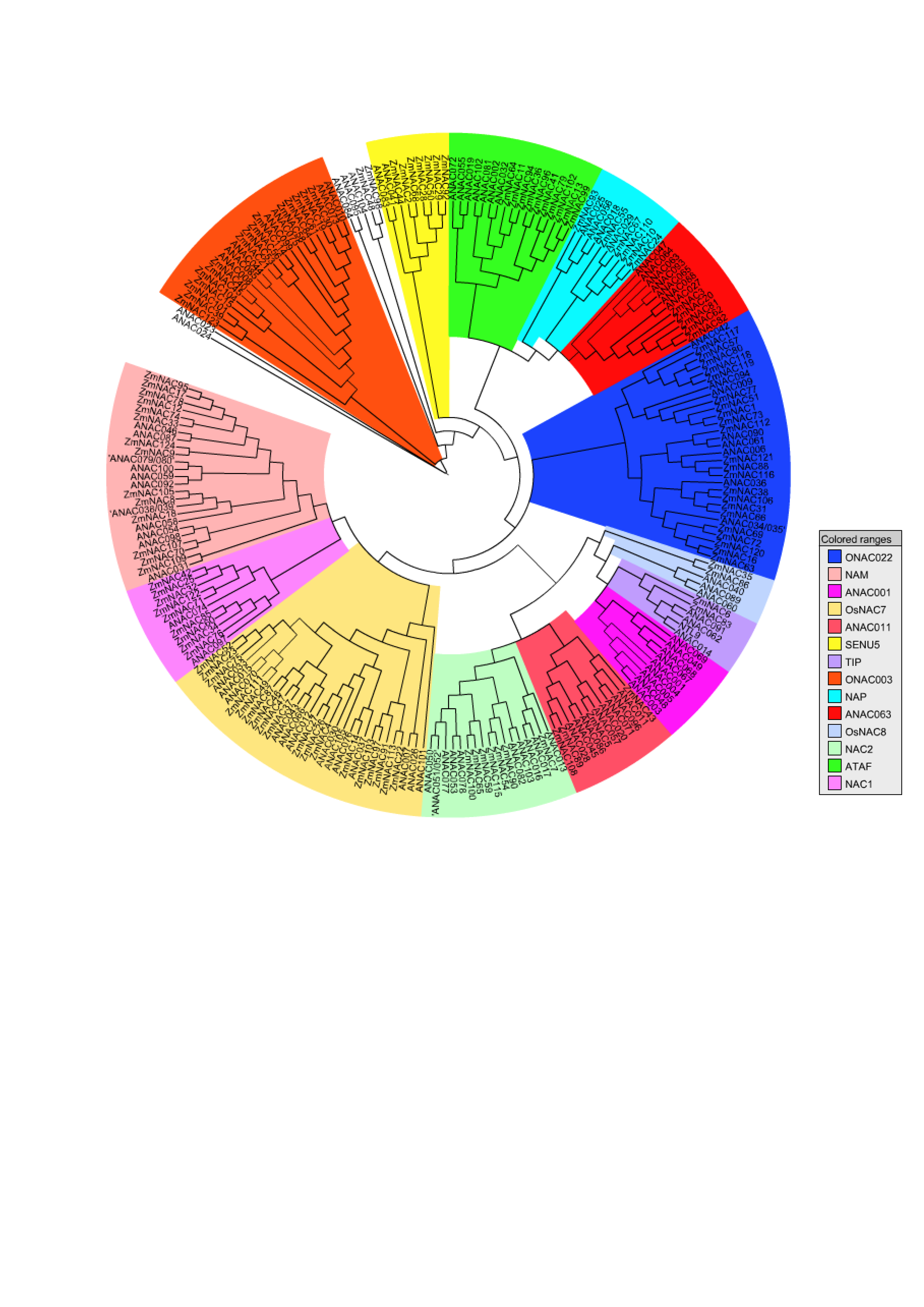

Supplement: Figure S2 — Phylogenetic tree of NAC proteins from Z. mays and Arabidopsis. Amino acid sequences were aligned using ClustalW and the maximum likelihood was generated through PhyML software. Names beginning with “ZmNAC” are NAC domains in Z. mays. All of ANACs in A. thaliana were achieved from TAIR. The NAC proteins of Z. mays are isolated as listed in . The subfamilies within the NAC family, as designated by Ooka et al (2003), are grouped by colors. (TIF) [file pone.0111837.s002.tif]

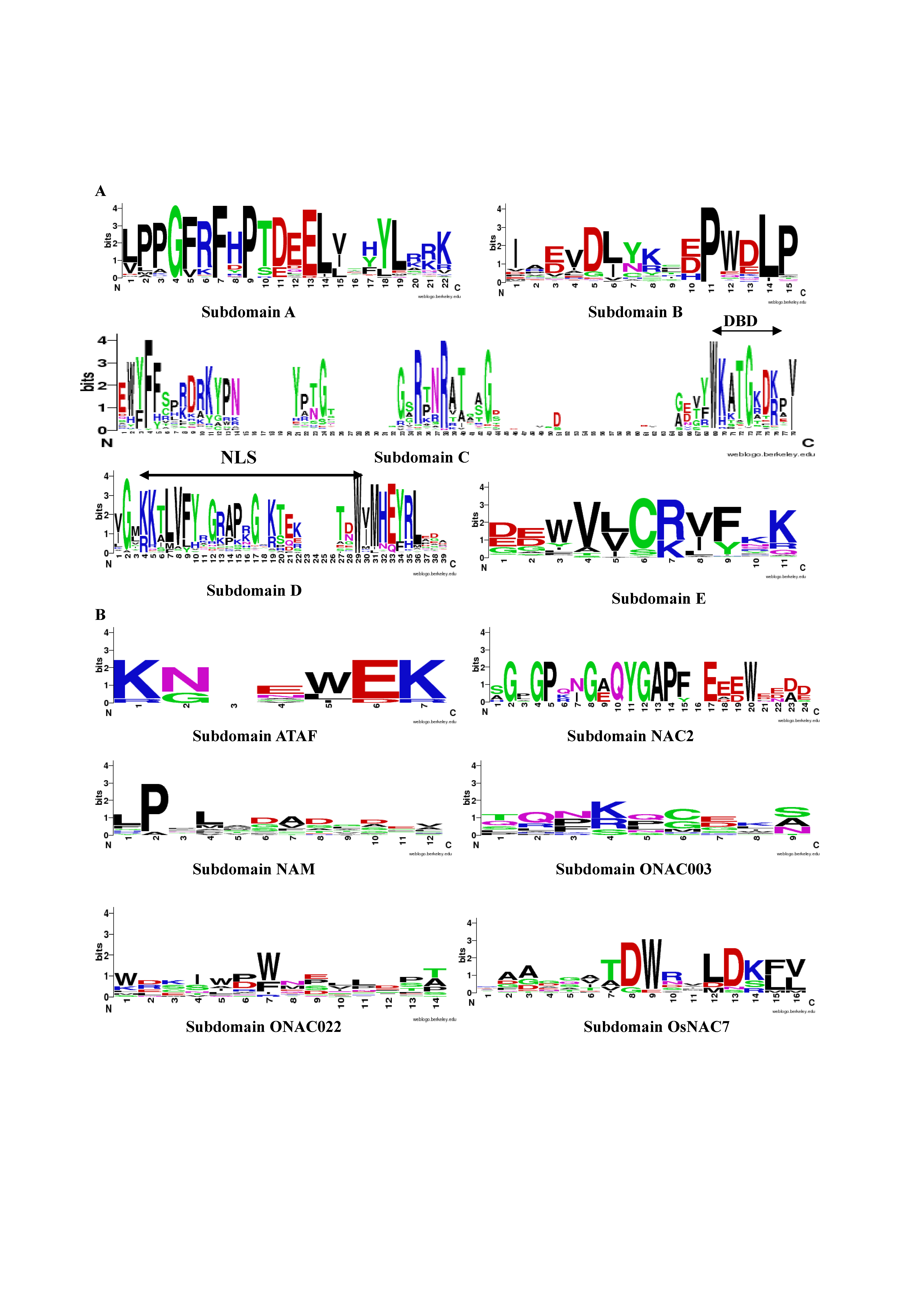

Supplement: Figure S3 — The conserved domain analysis in ZmNAC protein using WebLogo program. Sequence logos of NAC domain (A) and TAR region (B) among ZmNAC family. The height of letter designating the amino acid residue at each position represents the degree of conservation. The numbers on the x-axis represent the sequence positions in its corresponding conservative domains. The y-axis represents the information content measured in bits. (TIF) [file pone.0111837.s003.tif]

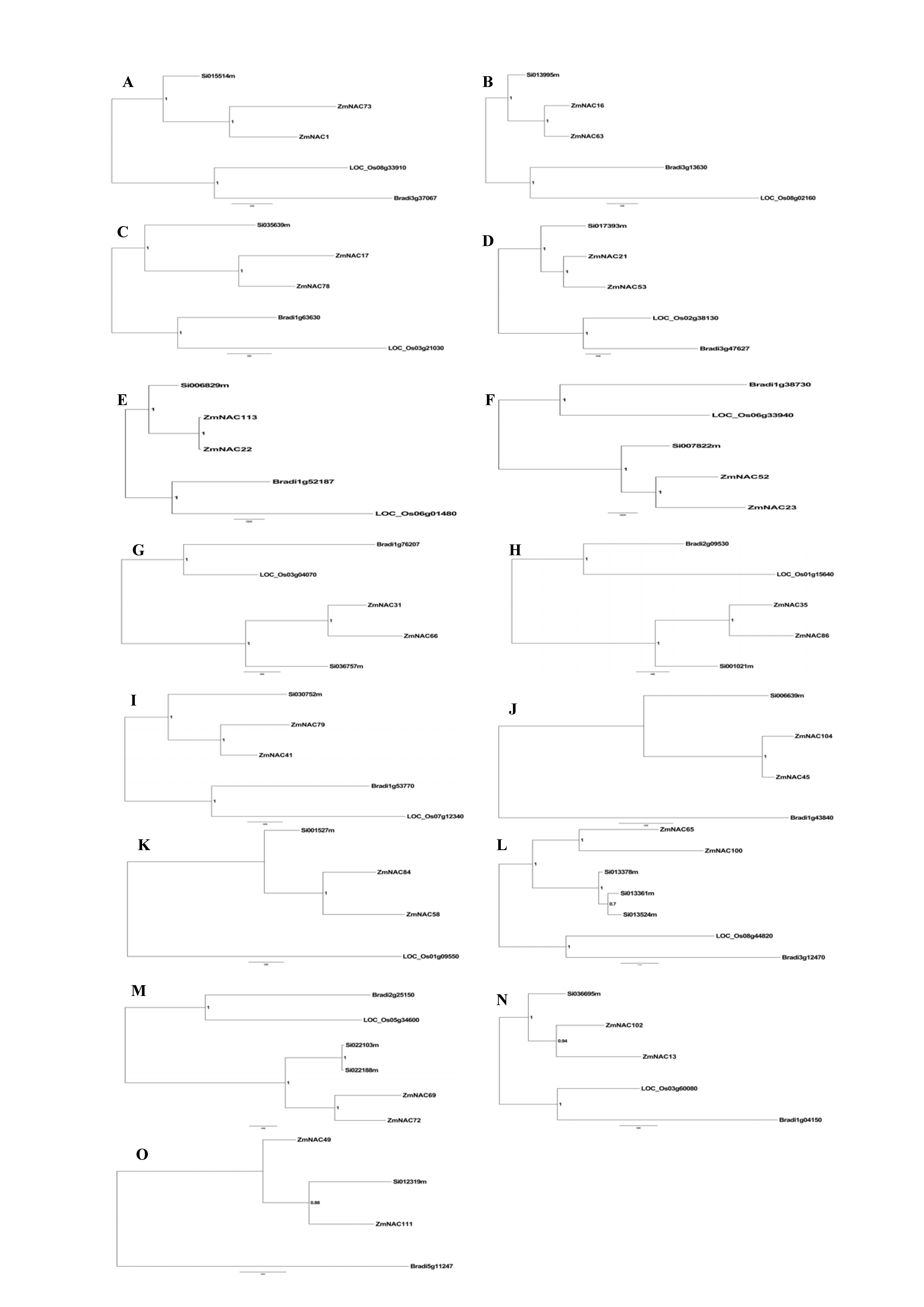

Supplement: Figure S4 — Phylogenetic relationships among the 15 pairs of duplicated ZmNAC genes and its corresponding orthologous genes in other three monocots. The Bayesian method was used to construct the phylogenetic tree. The numbers in the clades are posterior probability values. (TIF) [file pone.0111837.s004.tif]

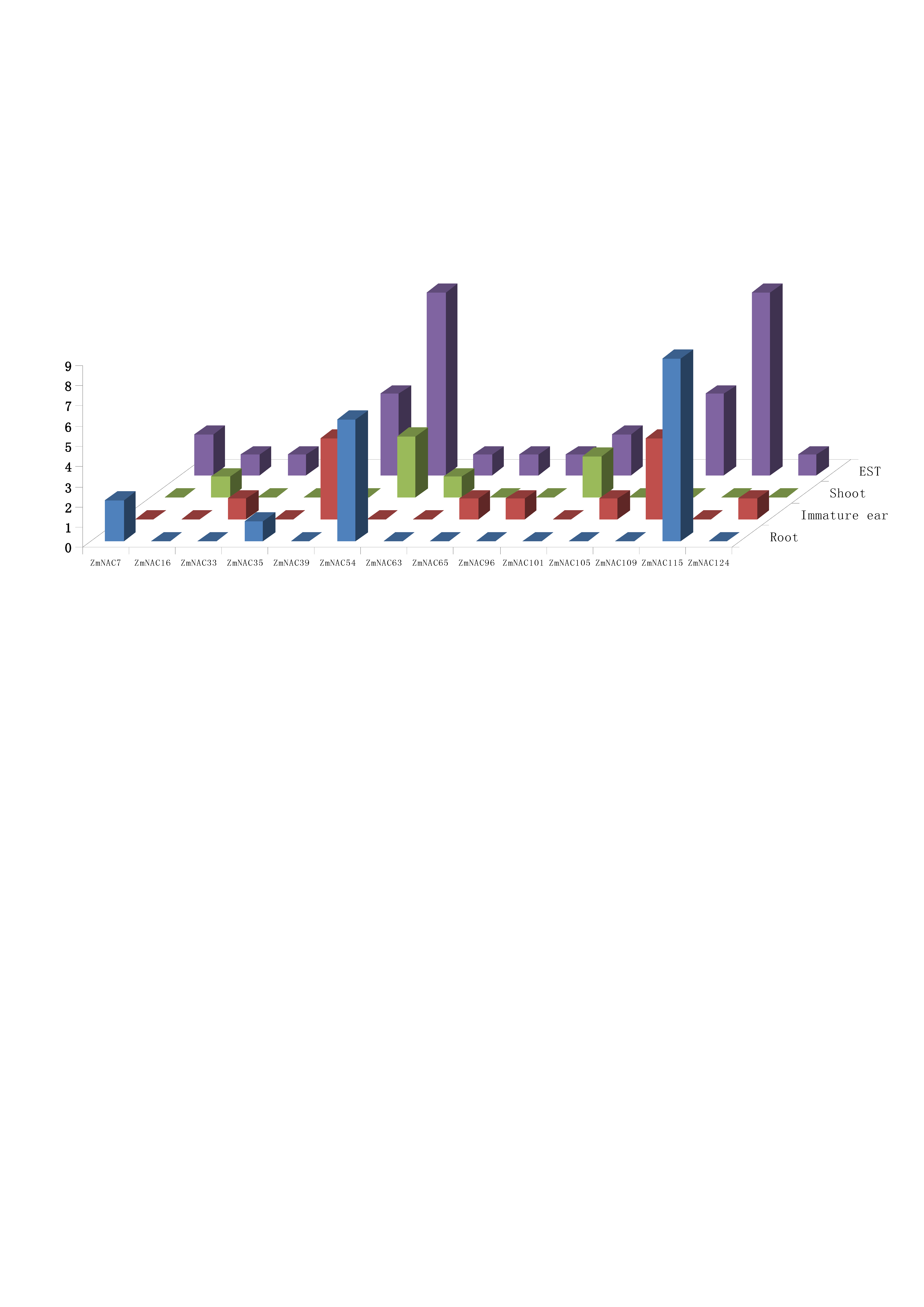

Supplement: Figure S5 — In silico frequency of Z. mays NAC genes ESTs. The EST frequencies of 14 ZmNAC genes were calculated through screening three EST libraries representing three different tissues. (TIF) [file pone.0111837.s005.tif]
