# Supplementary material for: Molecular Evolution and Expansion Analysis of the NAC Transcription Factor in Zea mays
Source: PLoS One. 2014 Nov 4;9(11):e111837. doi: 10.1371/journal.pone.0111837 (PMC4219692; doi:10.1371/journal.pone.0111837)
Supplement: Table S1 — ZmNAC proteins identified in Z. mays . (PDF) [file pone.0111837.s006.pdf]

Table S1. ZmNAC proteins identified in *Z. mays*.

Name	Locus name	Subfamily	Arabidopsis ortholog	E value	Arabidopsis locus description
ZmNAC1	AC198937.4_FG005	ONAC022	AT1G26870.1	3.00E-104	FEZ, ANAC009
ZmNAC2	AC203535.4_FG002	SENU5	AT3G04070.1	3.00E-25	ANAC047, NAC047
ZmNAC3	AC208663.3_FG002	ANAC063	AT5G22380.1	5.9	ANAC090, NAC090
ZmNAC4	AC212859.3_FG008	OsNAC7	AT1G71930.1	4.00E-88	VND7, ANAC030
ZmNAC5	AC233865.1_FG003	ANAC011	AT3G17730.1	3.00E-117	ANAC057, NAC057
ZmNAC6	GRMZM2G003715	TIP	AT1G33060.1	1.00E-63	ANAC014, NAC014
ZmNAC7	GRMZM2G004531	NAC2	AT1G34190.1	2.00E-64	ANAC017, NAC017
ZmNAC8	GRMZM2G008374	NAM	AT2G24430.1	2.00E-92	ANAC038,ANAC039
ZmNAC9	GRMZM2G009892	NAM	AT5G61430.1	7.00E-73	ANAC100, ATNAC5, NAC100
ZmNAC10	GRMZM2G011598	NAP	AT1G61110.1	2.00E-81	ANAC025, NAC025
ZmNAC11	GRMZM2G014653	ATAF	AT1G01720.1	4.00E-117	ATAF1, ANAC002
ZmNAC12	GRMZM2G018436	NAM	AT5G18270.1	2.00E-72	ANAC087
ZmNAC13	GRMZM2G018553	ATAF	AT5G08790.1	6.00E-83	ATAF2, ANAC081
ZmNAC14	GRMZM2G025642	OsNAC7	AT2G18060.1	4.00E-123	VND1, ANAC037
ZmNAC15	GRMZM2G027309	ONAC003	AT1G25580.1	2.00E-141	SOG1, ANAC008
ZmNAC16	GRMZM2G030325	ONAC022	AT2G02450.1	3.00E-94	ANAC034,ANAC035
ZmNAC17	GRMZM2G031001	NAM	AT5G18270.1	2.00E-87	ANAC087
ZmNAC18	GRMZM2G031120	NAM	AT3G18400.1	3.00E-90	ANAC058, NAC058
ZmNAC19	GRMZM2G031200	ONAC003	AT4G28500.1	4.00E-116	ANAC073, SND2, NAC073
ZmNAC20	GRMZM2G033014	ANAC063	AT3G15500.1	2.00E-07	ANAC055,ATNAC3
ZmNAC21	GRMZM2G038073	ONAC003	AT3G01600.1	2.00E-75	ANAC044, NAC044
ZmNAC22	GRMZM2G041668	OsNAC7	AT1G12260.1	4.00E-109	VND4, EMB2749, ANAC007, NAC007
ZmNAC23	GRMZM2G041746	OsNAC7	AT1G79580.1	1.00E-108	ANAC033, SMB

ZmNAC24	GRMZM2G042494	NAP	AT1G69490.1	2.00E-81	NAP, ANAC029, ATNAP
ZmNAC25	GRMZM2G043813	NAC1	AT4G28530.1	5.00E-75	ANAC074, NAC074
ZmNAC26	GRMZM2G048826	OsNAC7	AT1G71930.1	3.00E-79	VND7, ANAC030
ZmNAC27	GRMZM2G052239	OsNAC7	AT2G18060.1	6.00E-79	VND1, ANAC037
ZmNAC28	GRMZM2G054252	SENU5	AT1G52880.1	4.00E-35	ANAC018,ATNAM
ZmNAC29	GRMZM2G054277	SENU5	AT2G33480.1	3.00E-45	ANAC041
ZmNAC30	GRMZM2G058518	ONAC003	AT4G28500.1	7.00E-119	ANAC073, SND2, NAC073
ZmNAC31	GRMZM2G059428	ONAC022	AT2G17040.1	5.00E-81	ANAC036, NAC036
ZmNAC32	GRMZM2G062009	NAC1	AT4G28530.1	6.00E-73	ANAC074, NAC074
ZmNAC33	GRMZM2G062650	NAM	AT5G61430.1	5.00E-69	ANAC100, ATNAC5, NAC100
ZmNAC34	GRMZM2G063522	NAC1	AT3G12977.1	4.00E-78	-
ZmNAC35	GRMZM2G064541	OsNAC8	AT5G17260.1	3.00E-61	ANAC086
ZmNAC36	GRMZM2G068973	ATAF	AT5G08790.1	6.00E-79	ATAF2, ANAC081
ZmNAC37	GRMZM2G069047	OsNAC7	AT2G46770.1	3.00E-107	NST1, EMB2301, ANAC043
ZmNAC38	GRMZM2G074358	ONAC022	AT2G17040.1	3.00E-75	ANAC036, NAC036
ZmNAC39	GRMZM2G077045	ONAC003	AT1G25580.1	1.00E-32	SOG1, ANAC008
ZmNAC40	GRMZM2G078954	ONAC003	AT1G25580.1	1.00E-108	SOG1, ANAC008
ZmNAC41	GRMZM2G079632	ATAF	AT5G08790.1	8.00E-79	ATAF2, ANAC081
ZmNAC42	GRMZM2G081930	NAC1	AT4G28530.1	2.00E-79	ANAC074, NAC074
ZmNAC43	GRMZM2G082709	ANAC011	AT4G17980.1	4.00E-79	ANAC071, NAC071
ZmNAC44	GRMZM2G083347	SENU5	AT5G13180.1	4.00E-50	ANAC083, VNI2, NAC083
ZmNAC45	GRMZM2G086768	ONAC003	AT1G25580.1	2.00E-110	SOG1, ANAC008
ZmNAC46	GRMZM2G091490	OsNAC7	AT2G46770.1	2.00E-91	NST1, EMB2301, ANAC043
ZmNAC47	GRMZM2G092465	OsNAC7	AT2G46770.1	2.00E-104	NST1, EMB2301, ANAC043
ZmNAC48	GRMZM2G094067	No group	AT5G64530.1	1.00E-57	ANAC104, XND1
ZmNAC49	GRMZM2G099144	OsNAC7	AT4G10350.1	7.00E-109	ANAC070, BRN2, NAC070

ZmNAC50	GRMZM2G100583	SENU5	AT1G52880.1	2.00E-32	ANAC018,ATNAM
ZmNAC51	GRMZM2G100593	ONAC022	AT1G26870.1	9.00E-111	FEZ, ANAC009
ZmNAC52	GRMZM2G104074	OsNAC7	AT1G79580.1	4.00E-115	ANAC033, SMB
ZmNAC53	GRMZM2G104078	ONAC003	AT3G01600.1	2.00E-80	ANAC044, NAC044
ZmNAC54	GRMZM2G104400	NAC2	AT5G09330.1	4.00E-79	ANAC082,VNI1
ZmNAC55	GRMZM2G109627	NAP	AT3G04070.1	6.00E-77	ANAC047, NAC047
ZmNAC56	GRMZM2G111770	ONAC003	AT1G25580.1	1.00E-53	SOG1, ANAC008
ZmNAC57	GRMZM2G112548	ONAC022	AT2G43000.1	8.00E-81	ANAC042, NAC042
ZmNAC58	GRMZM2G112681	ONAC003	AT4G29230.1	1.00E-145	ANAC075, NAC075
ZmNAC59	GRMZM2G113950	NAC2	AT5G04410.1	6.00E-64	NAC2, ANAC078
ZmNAC60	GRMZM2G114850	NAC1	AT3G12977.1	2.00E-69	-
ZmNAC61	GRMZM2G115721	ONAC003	AT4G29230.1	5.00E-100	ANAC075, NAC075
ZmNAC62	GRMZM2G122615	ANAC063	AT2G43000.1	2.00E-17	ANAC042, NAC042
ZmNAC63	GRMZM2G123246	ONAC022	AT2G02450.1	2.00E-96	ANAC034,ANAC035
ZmNAC64	GRMZM2G123667	ATAF	AT5G08790.1	3.00E-91	ATAF2, ANAC081
ZmNAC65	GRMZM2G125777	NAC2	AT5G04410.1	8.00E-83	NAC2, ANAC078
ZmNAC66	GRMZM2G126817	ONAC022	AT2G17040.1	1.00E-81	ANAC036, NAC036
ZmNAC67	GRMZM2G127379	NAP	AT3G15510.1	2.00E-71	ATNAC2, ANAC056, NARS1, NAC2
ZmNAC68	GRMZM2G134073	SENU5	AT3G15510.1	9.00E-25	ATNAC2, ANAC056, NARS1, NAC2
ZmNAC69	GRMZM2G134687	ONAC022	AT2G02450.1	3.00E-95	ANAC034,ANAC035
ZmNAC70	GRMZM2G139700	NAM	AT5G53950.1	4.00E-89	CUC2, ANAC098, ATCUC2
ZmNAC71	GRMZM2G140901	NAC1	AT4G28530.1	6.00E-76	ANAC074, NAC074
ZmNAC72	GRMZM2G147867	ONAC022	AT2G02450.1	3.00E-95	ANAC034,ANAC035
ZmNAC73	GRMZM2G152543	ONAC022	AT1G26870.1	3.00E-107	FEZ, ANAC009
ZmNAC74	GRMZM2G154182	NAM	AT5G18270.1	1.00E-73	ANAC087
ZmNAC75	GRMZM2G155816	OsNAC7	AT1G79580.1	2.00E-82	ANAC033, SMB

ZmNAC76	GRMZM2G156977	NAC1	AT3G12977.1	2.00E-70	-
ZmNAC77	GRMZM2G159094	ONAC022	AT1G26870.1	1.00E-100	FEZ, ANAC009
ZmNAC78	GRMZM2G159500	NAM	AT5G18270.1	6.00E-93	ANAC087
ZmNAC79	GRMZM2G162739	ATAF	AT5G08790.1	1.00E-72	ATAF2, ANAC081
ZmNAC80	GRMZM2G163251	ONAC022	AT2G43000.1	7.00E-78	ANAC042, NAC042
ZmNAC81	GRMZM2G163841	ANAC063	AT5G08790.1	4.00E-13	ATAF2, ANAC081
ZmNAC82	GRMZM2G163843	ANAC063	AT1G26870.1	2.00E-17	FEZ, ANAC009
ZmNAC83	GRMZM2G163914	TIP	AT4G35580.1	1.00E-61	NTL9
ZmNAC84	GRMZM2G166721	ONAC003	AT4G29230.1	5.00E-148	ANAC075, NAC075
ZmNAC85	GRMZM2G167018	NAC1	AT3G12977.1	2.00E-69	-
ZmNAC86	GRMZM2G167492	OsNAC8	AT5G17260.1	2.00E-62	ANAC086
ZmNAC87	GRMZM2G171395	OsNAC7	AT2G46770.1	7.00E-94	NST1, EMB2301, ANAC043
ZmNAC88	GRMZM2G172264	ONAC022	AT5G22380.1	7.00E-56	ANAC090, NAC090
ZmNAC89	GRMZM2G174070	ANAC011	AT1G65910.1	9.00E-126	ANAC028, NAC028
ZmNAC90	GRMZM2G176677	NAC2	AT5G09330.1	4.00E-78	ANAC082,VNI1
ZmNAC91	GRMZM2G178998	OsNAC7	AT1G12260.1	1.00E-126	VND4, EMB2749, ANAC007, NAC007
ZmNAC92	GRMZM2G179049	SENU5	AT2G33480.1	9.00E-38	ANAC041
ZmNAC93	GRMZM2G179885	NAP	AT3G15510.1	6.00E-98	ATNAC2, ANAC056, NARS1, NAC2
ZmNAC94	GRMZM2G180328	ATAF	AT1G01720.1	7.00E-113	ATAF1, ANAC002
ZmNAC95	GRMZM2G181605	NAM	AT5G18270.1	6.00E-81	ANAC087
ZmNAC96	GRMZM2G312201	ATAF	AT5G08790.1	2.00E-76	ATAF2, ANAC081
ZmNAC97	GRMZM2G315140	OsNAC7	AT1G12260.1	9.00E-120	VND4, EMB2749, ANAC007, NAC007
ZmNAC98	GRMZM2G316840	No group	AT5G64530.1	2.00E-57	ANAC104, XND1
ZmNAC99	GRMZM2G336533	ATAF	AT5G08790.1	5.00E-82	ATAF2, ANAC081
ZmNAC100	GRMZM2G340305	NAC2	AT5G04410.1	6.00E-80	NAC2, ANAC078
ZmNAC101	GRMZM2G342647	ONAC003	AT1G25580.1	1.00E-31	SOG1, ANAC008

ZmNAC102	GRMZM2G347043	ATAF	AT5G08790.1	1.00E-80	ATAF2, ANAC081
ZmNAC103	GRMZM2G354151	OsNAC7	AT1G12260.1	2.00E-111	VND4, EMB2749, ANAC007, NAC007
ZmNAC104	GRMZM2G379608	ONAC003	AT1G25580.1	2.00E-85	SOG1, ANAC008
ZmNAC105	GRMZM2G386163	NAM	AT2G24430.1	5.00E-87	ANAC038,ANAC039
ZmNAC106	GRMZM2G389557	ONAC022	AT2G17040.1	1.00E-75	ANAC036, NAC036
ZmNAC107	GRMZM2G393433	NAM	AT5G53950.1	2.00E-85	CUC2, ANAC098, ATCUC2
ZmNAC108	GRMZM2G406204	ANAC011	AT1G65910.1	5.00E-130	ANAC028, NAC028
ZmNAC109	GRMZM2G430522	NAM	AT1G76420.1	2.00E-87	CUC3, NAC368, ANAC031
ZmNAC110	GRMZM2G430849	NAP	AT1G69490.1	2.00E-78	NAP, ANAC029, ATNAP
ZmNAC111	GRMZM2G435824	OsNAC7	AT4G10350.1	3.00E-100	ANAC070, BRN2, NAC070
ZmNAC112	GRMZM2G439903	ONAC022	AT5G22380.1	4.00E-58	ANAC090, NAC090
ZmNAC113	GRMZM2G440219	OsNAC7	AT1G12260.1	4.00E-109	VND4, EMB2749, ANAC007, NAC007
ZmNAC114	GRMZM2G450445	ONAC003	AT1G25580.1	6.00E-68	SOG1, ANAC008
ZmNAC115	GRMZM2G456568	NAC2	AT5G09330.1	4.00E-80	ANAC082,VNI1
ZmNAC116	GRMZM2G465835	ONAC022	AT5G22380.1	6.00E-58	ANAC090, NAC090
ZmNAC117	GRMZM2G475014	ONAC022	AT2G43000.1	7.00E-62	ANAC042, NAC042
ZmNAC118	GRMZM2G479980	ONAC022	AT2G43000.1	2.00E-75	ANAC042, NAC042
ZmNAC119	GRMZM5G803888	ONAC022	AT2G43000.1	3.00E-42	ANAC042, NAC042
ZmNAC120	GRMZM5G813651	ONAC022	AT2G02450.1	4.00E-103	ANAC034,ANAC035
ZmNAC121	GRMZM5G832473	ONAC022	AT5G22380.1	3.00E-61	ANAC090, NAC090
ZmNAC122	GRMZM5G857701	NAC1	AT4G28530.1	3.00E-75	ANAC074, NAC074
ZmNAC123	GRMZM5G894234	ONAC003	AT1G25580.1	2.00E-34	SOG1, ANAC008
ZmNAC124	GRMZM5G898290	NAM	AT5G07680.1	6.00E-82	ANAC079,ANAC080
