# Supplementary material for: Molecular Evolution and Expansion Analysis of the NAC Transcription Factor in Zea mays
Source: PLoS One. 2014 Nov 4;9(11):e111837. doi: 10.1371/journal.pone.0111837 (PMC4219692; doi:10.1371/journal.pone.0111837)
Supplement: Table S2 — List of the orthologous groups of ZmNAC proteins through OtrhoMCL clustering. (PDF) [file pone.0111837.s007.pdf]

Table S2. List of the orthologous groups of ZmNAC proteins through OtrhoMCL clustering.

Name	Subfamily	Orthomcl_group	Seq_id_of_best_hit	E value_mantissa	E value_exponent	Percent_identity	Percent_match
ZmNAC1	ONAC022	NO_GROUP	osat|NP_001061889	1	-151	70	99
ZmNAC2	SENU5	NO_GROUP	osat|NP_001045256	2	-42	55	83
ZmNAC3	ANAC063	OG5_242610	osat|NP_001043848	3	-6	32	51
ZmNAC4	OsNAC7	OG5_140455	osat|NP_001060775	2	-91	60	95
ZmNAC5	ANAC011	OG5_139552	rcom|30172.m000206	1	-103	74	100
ZmNAC6	TIP	OG5_160072	osat|NP_001061034	1	-178	53	97
ZmNAC7	NAC2	OG5_160025	osat|NP_001063547	0	-181	57	100
ZmNAC8	NAM	OG5_150285	osat|NP_001063563	1	-134	66	95
ZmNAC9	NAM	OG5_164656	osat|NP_001052992	1	-114	61	100
ZmNAC10	NAP	OG5_177391	osat|NP_001049997	1	-110	61	99
ZmNAC11	ATAF	OG5_135169	osat|NP_001045016	1	-133	81	100
ZmNAC12	NAM	OG5_205308	osat|NP_001066035	4	-80	59	77
ZmNAC13	ATAF	OG5_135169	osat|NP_001051682	1	-124	76	97
ZmNAC14	OsNAC7	OG5_170428	osat|NP_001048831	1	-144	75	99
ZmNAC15	ONAC003	OG5_213021	rcom|29738.m001046	1	-123	55	99
ZmNAC16	ONAC022	NO_GROUP	osat|NP_001060841	1	-93	70	65
ZmNAC17	NAM	OG5_164511	osat|NP_001049994	1	-119	65	100
ZmNAC18	NAM	OG5_190207	osat|NP_001050691	1	-105	64	100
ZmNAC19	ONAC003	OG5_170647	osat|NP_001056323	1	-121	74	99
ZmNAC20	ANAC063	OG5_242610	osat|NP_001043848	1	-24	38	57
ZmNAC21	ONAC003	OG5_177766	osat|NP_001047308	1	-157	71	100
ZmNAC22	OsNAC7	OG5_140455	osat|NP_001056549	1	-133	71	95
ZmNAC23	OsNAC7	OG5_160167	osat|NP_001046449	1	-111	59	99
ZmNAC24	NAP	OG5_177391	osat|NP_001049997	9	-95	72	100
ZmNAC25	NAC1	OG5_170224	osat|NP_001053312	1	-100	68	100
ZmNAC26	OsNAC7	OG5_140455	osat|NP_001060775	3	-92	60	94
ZmNAC27	OsNAC7	OG5_140455	osat|NP_001060775	5	-84	74	75

ZmNAC28	SENU5	OG5_243089	osat|NP_001062358	1	-46	59	81
ZmNAC29	SENU5	OG5_243089	osat|NP_001063626	1	-48	58	96
ZmNAC30	ONAC003	OG5_170647	rcom|29983.m003125	1	-112	63	99
ZmNAC31	ONAC022	OG5_178238	osat|NP_001048872	1	-116	72	99
ZmNAC32	NAC1	OG5_170224	osat|NP_001053312	1	-87	73	75
ZmNAC33	NAM	OG5_242540	osat|NP_001041765	3	-64	42	99
ZmNAC34	NAC1	OG5_164225	osat|NP_001058346	1	-135	77	100
ZmNAC35	OsNAC8	NO_GROUP	osat|NP_001042648	1	-133	51	97
ZmNAC36	ATAF	OG5_135169	osat|NP_001044617	1	-123	74	100
ZmNAC37	OsNAC7	OG5_170479	rcom|27964.m000369	2	-97	54	92
ZmNAC38	ONAC022	OG5_178238	osat|NP_001058628	1	-104	64	93
ZmNAC39	ONAC003	OG5_128977	osat|NP_001058575	2	-27	41	54
ZmNAC40	ONAC003	OG5_213021	rcom|29738.m001046	1	-104	54	82
ZmNAC41	ATAF	OG5_135169	osat|NP_001059213	2	-98	70	99
ZmNAC42	NAC1	OG5_170224	osat|NP_001053312	1	-107	69	100
ZmNAC43	ANAC011	OG5_190411	rcom|29827.m002555	1	-74	45	100
ZmNAC44	SENU5	NO_GROUP	osat|NP_001066757	1	-78	83	68
ZmNAC45	ONAC003	OG5_213021	rcom|29738.m001046	5	-96	69	76
ZmNAC46	OsNAC7	OG5_170479	rcom|27964.m000369	1	-87	50	93
ZmNAC47	OsNAC7	OG5_170479	osat|NP_001060852	1	-103	77	93
ZmNAC48	-	OG5_178154	osat|NP_001047122	1	-75	68	100
ZmNAC49	OsNAC7	OG5_212584	atha|NP_192773	2	-90	50	99
ZmNAC50	SENU5	OG5_243089	osat|NP_001062358	3	-48	62	79
ZmNAC51	ONAC022	OG5_177548	osat|NP_001048109	0	-181	82	100
ZmNAC52	OsNAC7	OG5_160167	osat|NP_001046449	1	-110	60	99
ZmNAC53	ONAC003	OG5_177766	osat|NP_001047308	1	-150	67	100
ZmNAC54	NAC2	OG5_156359	osat|NP_001055600	1	-132	55	100
ZmNAC55	NAP	OG5_177391	osat|NP_001049997	1	-77	56	70
ZmNAC56	ONAC003	OG5_177766	osat|NP_001047308	3	-73	43	93

ZmNAC57	ONAC022	OG5_190119	osat|NP_001051438	1	-110	63	100
ZmNAC58	ONAC003	OG5_190453	osat|NP_001042270	0	-181	81	94
ZmNAC59	NAC2	OG5_139552	osat|NP_001048559	0	-181	62	100
ZmNAC60	NAC1	NO_GROUP	osat|NP_001067248	1	-102	63	99
ZmNAC61	ONAC003	OG5_190453	osat|NP_001042270	7	-87	52	90
ZmNAC62	ANAC063	OG5_242610	osat|NP_001043848	7	-31	44	53
ZmNAC63	ONAC022	NO_GROUP	osat|NP_001060841	2	-92	70	53
ZmNAC64	ATAF	NO_GROUP	osat|NP_001065919	1	-117	66	100
ZmNAC65	NAC2	OG5_139552	osat|NP_001062518	0	-181	77	97
ZmNAC66	ONAC022	OG5_178238	osat|NP_001048872	1	-110	68	99
ZmNAC67	NAP	OG5_243148	osat|NP_001066034	6	-95	49	100
ZmNAC68	SENU5	NO_GROUP	osat|NP_001045256	1	-37	53	83
ZmNAC69	ONAC022	OG5_164698	osat|NP_001055568	1	-106	60	91
ZmNAC70	NAM	OG5_150285	osat|NP_001057578	1	-103	63	96
ZmNAC71	NAC1	OG5_170224	osat|NP_001048472	2	-87	57	100
ZmNAC72	ONAC022	OG5_164698	osat|NP_001055568	1	-106	59	91
ZmNAC73	ONAC022	NO_GROUP	osat|NP_001061889	1	-149	68	99
ZmNAC74	NAM	OG5_242540	osat|NP_001041765	8	-68	46	96
ZmNAC75	OsNAC7	OG5_160167	osat|NP_001046449	1	-77	89	93
ZmNAC76	NAC1	NO_GROUP	osat|NP_001053895	2	-73	53	97
ZmNAC77	ONAC022	NO_GROUP	osat|NP_001061889	1	-112	55	93
ZmNAC78	NAM	OG5_164511	osat|NP_001049994	1	-124	65	100
ZmNAC79	ATAF	OG5_135169	osat|NP_001059213	2	-96	67	100
ZmNAC80	ONAC022	OG5_190119	osat|NP_001051438	1	-105	59	99
ZmNAC81	ANAC063	OG5_242610	osat|NP_001043848	3	-38	52	53
ZmNAC82	ANAC063	OG5_242610	osat|NP_001043848	3	-39	48	54
ZmNAC83	TIP	OG5_160072	osat|NP_001056531	1	-111	58	97
ZmNAC84	ONAC003	OG5_190453	osat|NP_001042270	0	-181	85	100
ZmNAC85	NAC1	OG5_164225	osat|NP_001061213	8	-87	58	98

ZmNAC86	OsNAC8	NO_GROUP	osat|NP_001042648	1	-125	49	97
ZmNAC87	OsNAC7	NO_GROUP	osat|NP_001056697	5	-92	76	100
ZmNAC88	ONAC022	OG5_156371	osat|NP_001055672	1	-78	57	99
ZmNAC89	ANAC011	OG5_139552	rcom|30171.m000407	1	-135	49	78
ZmNAC90	NAC2	OG5_156359	osat|NP_001055600	3	-75	43	90
ZmNAC91	OsNAC7	OG5_140455	rcom|28200.m000189	1	-108	58	100
ZmNAC92	SENU5	OG5_243089	osat|NP_001063626	4	-47	56	96
ZmNAC93	NAP	OG5_135169	osat|NP_001060017	1	-141	67	99
ZmNAC94	ATAF	OG5_135169	osat|NP_001045016	1	-116	68	99
ZmNAC95	NAM	OG5_164511	osat|NP_001060671	5	-83	57	93
ZmNAC96	ATAF	OG5_242640	osat|NP_001044616	0	-181	76	95
ZmNAC97	OsNAC7	OG5_140455	rcom|28200.m000189	1	-104	55	100
ZmNAC98	-	OG5_178154	osat|NP_001052856	2	-69	67	99
ZmNAC99	ATAF	OG5_135169	osat|NP_001051682	1	-124	76	97
ZmNAC100	NAC2	OG5_139552	osat|NP_001062518	0	-181	76	94
ZmNAC101	ONAC003	OG5_170647	atha|NP_194579	2	-26	34	55
ZmNAC102	ATAF	OG5_135169	osat|NP_001051682	1	-135	79	99
ZmNAC103	OsNAC7	OG5_140455	rcom|28200.m000189	3	-96	52	100
ZmNAC104	ONAC003	OG5_213021	rcom|29738.m001046	1	-78	71	70
ZmNAC105	NAM	OG5_150285	osat|NP_001063563	1	-133	71	93
ZmNAC106	ONAC022	OG5_178238	osat|NP_001058628	9	-94	68	96
ZmNAC107	NAM	OG5_150285	osat|NP_001057578	1	-110	69	96
ZmNAC108	ANAC011	OG5_139552	rcom|30171.m000407	1	-136	50	78
ZmNAC109	NAM	OG5_189953	osat|NP_001062212	1	-117	65	82
ZmNAC110	NAP	OG5_177391	osat|NP_001049997	8	-87	64	64
ZmNAC111	OsNAC7	OG5_212584	rcom|29382.m000084	2	-88	51	94
ZmNAC112	ONAC022	OG5_156371	osat|NP_001065785	4	-79	59	94
ZmNAC113	OsNAC7	OG5_140455	osat|NP_001056549	1	-133	71	95
ZmNAC114	ONAC003	OG5_177766	osat|NP_001047308	5	-79	50	78

ZmNAC115	NAC2	OG5_156359	osat|NP_001055600	1	-132	56	97
ZmNAC116	ONAC022	OG5_156371	osat|NP_001055672	4	-82	63	99
ZmNAC117	ONAC022	OG5_190119	rcom|30169.m006343	9	-62	61	58
ZmNAC118	ONAC022	OG5_190119	osat|NP_001051438	1	-93	56	94
ZmNAC119	ONAC022	OG5_190119	osat|NP_001051438	2	-47	70	51
ZmNAC120	ONAC022	OG5_164698	osat|NP_001045038	1	-126	65	94
ZmNAC121	ONAC022	OG5_156371	osat|NP_001044885	3	-82	63	96
ZmNAC122	NAC1	OG5_170224	osat|NP_001048472	4	-89	57	97
ZmNAC123	ONAC003	OG5_190453	rcom|29912.m005354	3	-30	34	52
ZmNAC124	NAM	OG5_164656	osat|NP_001047228	1	-105	61	96
