# Supplementary material for: Molecular Evolution and Expansion Analysis of the NAC Transcription Factor in Zea mays
Source: PLoS One. 2014 Nov 4;9(11):e111837. doi: 10.1371/journal.pone.0111837 (PMC4219692; doi:10.1371/journal.pone.0111837)
Supplement: Table S3 — The structural analysis of ZmNAC identified in this study. (PDF) [file pone.0111837.s008.pdf]

Table S3. The structural analysis of ZmNAC identified in this study.

Name	Extron number	Intron number	Molecular weight	Theoretical pI	protein length (aa)	α -helix	Extended strand	β -turn	Random coil
ZmNAC1	3	2	42497.7	7.68	381	74	49	13	245
ZmNAC2	3	2	26239.4	8.21	246	54	29	8	155
ZmNAC3	1	0	40789.1	9.15	362	118	59	27	158
ZmNAC4	3	2	35725.9	6.07	320	80	52	12	176
ZmNAC5	3	2	27690.0	5.33	245	65	36	9	135
ZmNAC6	7	6	73605.6	4.81	664	163	98	48	355
ZmNAC7	4	3	77981.6	4.67	709	245	89	52	323
ZmNAC8	3	2	45014.0	9.30	409	110	62	26	211
ZmNAC9	3	2	39040.6	6.87	365	133	45	31	156
ZmNAC10	3	2	40588.6	8.70	373	90	43	16	224
ZmNAC11	3	2	32544.2	8.46	295	58	33	14	190
ZmNAC12	3	2	35364.4	6.53	322	69	53	24	176
ZmNAC13	2	1	33831.2	6.56	302	84	37	11	170
ZmNAC14	3	2	39962.5	5.60	362	134	50	18	160
ZmNAC15	6	5	46759.1	5.30	413	113	66	22	212
ZmNAC16	3	2	41837.0	9.36	380	82	46	25	227
ZmNAC17	3	2	47600.3	5.87	433	156	58	22	197
ZmNAC18	2	1	35900.6	7.14	326	88	51	15	172
ZmNAC19	3	2	34619.0	9.22	319	90	57	23	149
ZmNAC20	1	0	33779.7	8.59	315	51	56	15	193
ZmNAC21	6	5	44936.4	5.10	399	96	58	21	224
ZmNAC22	3	2	40215.9	6.46	349	64	46	7	232
ZmNAC23	3	2	41425.8	6.43	369	94	47	22	206
ZmNAC24	3	2	26619.3	9.08	241	50	28	6	157

ZmNAC25	2	1	32395.3	5.68	293	49	43	12	189
ZmNAC26	2	1	33128.9	6.26	295	47	38	10	200
ZmNAC27	2	1	31005.5	6.59	277	59	55	10	153
ZmNAC28	3	2	24712.3	10.52	231	40	42	13	136
ZmNAC29	3	2	22837.2	11.17	202	40	29	10	123
ZmNAC30	3	2	35357.5	8.96	323	100	54	14	155
ZmNAC31	3	2	35641.1	6.72	326	88	53	18	166
ZmNAC32	3	2	31825.3	6.15	294	40	55	23	176
ZmNAC33	3	2	42132.1	5.81	391	72	83	28	208
ZmNAC34	3	2	34128.7	8.17	305	79	44	15	167
ZmNAC35	4	3	57502.0	5.49	517	103	86	30	298
ZmNAC36	3	2	34512.0	8.79	308	82	40	15	171
ZmNAC37	3	2	41286.3	8.55	379	140	55	25	159
ZmNAC38	2	1	36590.3	6.33	326	82	54	11	179
ZmNAC39	14	13	59811.3	5.73	526	245	70	46	165
ZmNAC40	5	4	73548.0	9.04	672	149	95	46	382
ZmNAC41	2	1	33628.7	5.31	300	91	39	10	160
ZmNAC42	3	2	32549.4	5.62	297	53	57	20	167
ZmNAC43	4	3	37371.2	5.56	343	114	54	23	152
ZmNAC44	2	1	27865.5	10.08	259	53	37	14	155
ZmNAC45	5	4	34225.5	7.26	303	41	65	19	178
ZmNAC46	3	2	39410.1	6.59	367	119	49	25	174
ZmNAC47	3	2	43953.5	6.44	418	173	46	32	167
ZmNAC48	3	2	24476.0	6.10	225	36	40	16	133
ZmNAC49	3	2	39900.4	7.32	362	91	69	46	156
ZmNAC50	2	1	24313.9	10.46	228	37	38	11	142

ZmNAC51	3	2	43475.2	8.97	386	65	37	9	275
ZmNAC52	3	2	40012.3	6.36	357	107	40	14	196
ZmNAC53	6	5	44949.7	5.67	399	106	57	18	218
ZmNAC54	4	3	49513.1	4.61	445	89	52	24	280
ZmNAC55	3	2	42481.3	6.18	398	105	66	18	209
ZmNAC56	5	4	47660.3	5.55	438	128	62	22	226
ZmNAC57	3	2	37285.1	8.48	336	73	42	14	207
ZmNAC58	6	5	54977.5	7.04	494	122	76	24	272
ZmNAC59	5	4	71976.4	4.62	657	127	74	14	442
ZmNAC60	3	2	36137.3	9.01	338	108	47	23	160
ZmNAC61	5	4	38729.8	6.40	354	85	54	28	187
ZmNAC62	1	0	36083.8	5.67	329	51	51	17	210
ZmNAC63	3	2	40610.9	9.31	368	92	45	18	213
ZmNAC64	3	2	38842.4	5.25	359	88	51	24	196
ZmNAC65	5	4	71824.7	4.53	665	111	98	34	422
ZmNAC66	3	2	34853.3	6.21	317	101	51	14	151
ZmNAC67	3	2	51243.5	5.82	476	88	105	23	259
ZmNAC68	3	2	27215.5	8.71	259	40	37	11	171
ZmNAC69	3	2	45293.8	6.37	425	130	45	28	222
ZmNAC70	3	2	38726.9	8.72	368	47	74	30	217
ZmNAC71	2	1	32555.3	6.20	298	54	49	14	181
ZmNAC72	3	2	45153.0	8.35	420	121	38	27	234
ZmNAC73	3	2	43377.7	8.19	392	45	55	6	286
ZmNAC74	3	2	38523.7	6.91	359	83	54	12	210
ZmNAC75	2	1	18646.1	9.22	160	14	34	7	105
ZmNAC76	3	2	34542.9	7.12	317	70	43	18	186

ZmNAC77	4	3	46747.6	8.56	419	78	63	21	257
ZmNAC78	3	2	38431.3	5.71	348	82	48	12	206
ZmNAC79	2	1	33893.1	5.74	303	110	44	8	141
ZmNAC80	3	2	40721.8	7.66	368	113	45	23	187
ZmNAC81	1	0	34295.1	5.94	312	88	50	18	156
ZmNAC82	1	0	38206.0	5.52	356	76	45	18	217
ZmNAC83	6	5	66805.6	5.58	612	181	87	25	319
ZmNAC84	6	5	48585.4	7.75	436	108	63	22	243
ZmNAC85	3	2	31172.4	7.08	282	59	46	11	166
ZmNAC86	4	3	57331.8	5.52	518	96	78	24	320
ZmNAC87	3	2	47060.4	6.25	435	162	76	34	163
ZmNAC88	3	2	31152.3	5.65	283	65	43	12	163
ZmNAC89	6	5	76138.6	5.88	687	196	88	30	373
ZmNAC90	4	3	44754.2	5.21	408	89	66	26	227
ZmNAC91	3	2	39686.6	6.17	344	87	40	8	209
ZmNAC92	3	2	30510.5	9.97	285	36	54	11	184
ZmNAC93	3	2	43556.8	6.92	410	95	65	15	235
ZmNAC94	3	2	35805.5	9.15	339	64	37	18	220
ZmNAC95	3	2	35343.9	6.83	318	79	33	9	197
ZmNAC96	8	7	163163.6	7.05	1467	799	139	124	405
ZmNAC97	3	2	38719.4	6.01	342	74	33	11	224
ZmNAC98	3	2	22834.1	4.93	211	43	38	11	119
ZmNAC99	2	1	48024.2	8.23	436	114	60	19	243
ZmNAC100	5	4	81959.4	4.79	763	147	141	45	430
ZmNAC101	9	8	63309.3	5.40	566	180	92	30	264
ZmNAC102	2	1	34841.3	6.27	312	96	40	7	169

ZmNAC103	3	2	42773.0	5.73	380	91	50	17	222
ZmNAC104	4	3	29987.7	6.39	264	39	67	17	141
ZmNAC105	9	8	98205.9	5.64	882	219	176	89	398
ZmNAC106	1	0	30078.1	9.28	268	54	40	8	166
ZmNAC107	3	2	37895.7	8.71	358	54	71	33	200
ZmNAC108	7	6	73381.2	5.60	664	166	106	35	357
ZmNAC109	2	1	46650.5	7.64	423	112	64	36	210
ZmNAC110	3	2	42079.7	6.89	395	99	45	17	234
ZmNAC111	4	3	38554.4	5.96	349	84	54	39	172
ZmNAC112	2	1	33841.7	6.34	309	73	57	20	159
ZmNAC113	3	2	40215.9	6.46	349	64	46	7	232
ZmNAC114	7	6	48176.4	5.81	438	116	51	30	241
ZmNAC115	4	3	50480.2	4.43	452	98	54	17	283
ZmNAC116	3	2	30229.5	6.75	272	65	35	10	162
ZmNAC117	3	2	43119.9	8.63	371	144	47	17	163
ZmNAC118	2	1	39965.5	5.80	358	86	45	21	206
ZmNAC119	2	1	27691.8	9.42	251	54	38	27	132
ZmNAC120	3	2	47279.3	8.98	447	131	59	34	223
ZmNAC121	3	2	31208.6	4.86	292	66	45	15	166
ZmNAC122	2	1	32497.4	6.67	296	61	54	15	166
ZmNAC123	7	6	40175.8	4.92	361	90	70	22	179
ZmNAC124	3	2	38019.1	6.67	348	85	47	16	200
