# Supplementary material for: Molecular Evolution and Expansion Analysis of the NAC Transcription Factor in Zea mays
Source: PLoS One. 2014 Nov 4;9(11):e111837. doi: 10.1371/journal.pone.0111837 (PMC4219692; doi:10.1371/journal.pone.0111837)
Supplement: Table S4 — Genomic locations of NAC genes in Z. mays . (PDF) [file pone.0111837.s009.pdf]

Table S4. Genomic locations of NAC genes in *Z. mays*.

ID	Chromosome	Start (bp)	End (bp)
ZmNAC1	04	83571704	83573094
ZmNAC2	03	157575853	157577265
ZmNAC3	02	29926105	29927193
ZmNAC4	02	25745084	25746893
ZmNAC5	07	146597172	146598863
ZmNAC6	10	78128657	78132883
ZmNAC7	07	134099371	134102423
ZmNAC8	02	190853955	190855836
ZmNAC9	02	41885688	41887591
ZmNAC10	01	53633788	53636032
ZmNAC11	03	170820346	170822801
ZmNAC12	02	150029400	150030906
ZmNAC13	05	2917535	2919211
ZmNAC14	01	5635814	5638956
ZmNAC15	06	116578564	116583279
ZmNAC16	06	3869561	3872846
ZmNAC17	01	53421632	53423835
ZmNAC18	01	254075089	254076572
ZmNAC19	06	164497332	164499048
ZmNAC20	07	2717208	2718616
ZmNAC21	05	180370272	180373773
ZmNAC22	09	28039764	28043748
ZmNAC23	06	106251283	106253155
ZmNAC24	09	132248666	132251004
ZmNAC25	10	130620219	130622989
ZmNAC26	04	59337242	59338351
ZmNAC27	06	779435	780648
ZmNAC28	01	195185126	195188536
ZmNAC29	07	135891455	135892601
ZmNAC30	03	210231409	210235812
ZmNAC31	01	7488692	7490758
ZmNAC32	04	142249356	142251782
ZmNAC33	03	31804638	31806658
ZmNAC34	05	43417416	43421787
ZmNAC35	03	38053287	38062585
ZmNAC36	08	170862648	170864431
ZmNAC37	04	38028298	38030761
ZmNAC38	06	82764321	82765776
ZmNAC39	01	25171943	25180175
ZmNAC40	06	116673745	116677264
ZmNAC41	07	20892397	20894232
ZmNAC42	02	29121394	29124256
ZmNAC43	01	98096125	98097592
ZmNAC44	10	14444468	14446346
ZmNAC45	06	116657254	116667015
ZmNAC46	06	66026862	66029306
ZmNAC47	06	4305832	4308433

ZmNAC48	05	172920167	172921326
ZmNAC49	02	47046908	47048343
ZmNAC50	04	46934197	46936520
ZmNAC51	05	206975367	206978937
ZmNAC52	09	64929704	64931633
ZmNAC53	04	130518600	130522290
ZmNAC54	08	102534874	102538745
ZmNAC55	08	7035985	7038701
ZmNAC56	10	125194444	125197616
ZmNAC57	05	5326928	5328393
ZmNAC58	08	20827673	20834211
ZmNAC59	04	170244250	170248670
ZmNAC60	03	122071793	122076122
ZmNAC61	09	55508378	55513309
ZmNAC62	03	209087673	209088984
ZmNAC63	04	38541622	38544681
ZmNAC64	04	207978208	207980920
ZmNAC65	04	50096485	50101420
ZmNAC66	09	151672368	151673873
ZmNAC67	10	2679313	2681402
ZmNAC68	08	160424732	160426914
ZmNAC69	08	103366829	103369759
ZmNAC70	03	137579039	137580848
ZmNAC71	04	172192989	172195242
ZmNAC72	06	147512405	147515068
ZmNAC73	01	212951664	212953388
ZmNAC74	08	7299739	7301739
ZmNAC75	05	142183507	142185261
ZmNAC76	02	9413823	9416383
ZmNAC77	07	111666002	111668186
ZmNAC78	09	132812862	132814990
ZmNAC79	02	158941564	158943462
ZmNAC80	01	283250286	283252184
ZmNAC81	08	150573802	150574767
ZmNAC82	08	150569499	150570962
ZmNAC83	09	28843767	28846916
ZmNAC84	03	6938407	6944754
ZmNAC85	10	61000097	61002745
ZmNAC86	08	4541745	4558891
ZmNAC87	09	23279027	23283429
ZmNAC88	08	99283137	99284291
ZmNAC89	09	153957735	153962180
ZmNAC90	02	26430610	26438582
ZmNAC91	02	22703231	22706465
ZmNAC92	02	192453409	192455615
ZmNAC93	07	158236773	158238970
ZmNAC94	06	147868257	147870276
ZmNAC95	07	173738865	173740690
ZmNAC96	03	186198585	186207556
ZmNAC97	05	189401545	189405925

ZmNAC98	02	48822571	48824076
ZmNAC99	05	2887052	2888841
ZmNAC100	01	203087262	203092166
ZmNAC101	08	20166676	20174312
ZmNAC102	01	292086460	292088246
ZmNAC103	04	148816480	148820387
ZmNAC104	06	116669547	116672514
ZmNAC105	07	134693895	134703415
ZmNAC106	05	63277675	63280116
ZmNAC107	06	16291162	16293314
ZmNAC108	01	4248315	4252340
ZmNAC109	01	188906106	188908967
ZmNAC110	07	173514759	173517298
ZmNAC111	10	119126027	119127985
ZmNAC112	04	189106362	189107549
ZmNAC113	09	28134439	28136186
ZmNAC114	02	37553208	37559085
ZmNAC115	06	147921021	147925447
ZmNAC116	06	149397669	149398803
ZmNAC117	01	178400843	178402328
ZmNAC118	07	4582968	4584136
ZmNAC119	02	236667599	236668913
ZmNAC120	03	170022873	170025674
ZmNAC121	03	176510723	176512231
ZmNAC122	05	214949731	214951374
ZmNAC123	09	146252099	146255808
ZmNAC124	04	125772509	125774241
