# Supplementary material for: Molecular Evolution and Expansion Analysis of the NAC Transcription Factor in Zea mays
Source: PLoS One. 2014 Nov 4;9(11):e111837. doi: 10.1371/journal.pone.0111837 (PMC4219692; doi:10.1371/journal.pone.0111837)
Supplement: Table S5 — The duplicated ZmNAC genes and its corresponding orthologous genes in other three monocots. (PDF) [file pone.0111837.s010.pdf]

Table S5. The duplicated ZmNAC genes and its corresponding orthologous genes in other three monocots.

Duplicated gene 1	Duplicated gene 2	Locus name of its corresponding orthologous gene in some monocots			Orthomcl_group
		Setaria italica	*Oryza sativa*	*Brachypodium distachyon*	
ZmNAC1	ZmNAC73	Si015514m	LOC_Os08g33910	Bradi3g37067	NO_GROUP
ZmNAC13	ZmNAC102	Si036695m	LOC_Os03g60080	Bradi1g04150	OG5_135169
ZmNAC16	ZmNAC63	Si013995m	LOC_Os08g02160	Bradi3g13630	NO_GROUP
ZmNAC17	ZmNAC78	Si035639m	LOC_Os03g21030	Bradi1g63630	OG5_164511
ZmNAC21	ZmNAC53	Si017393m	LOC_Os02g38130	Bradi3g47627	OG5_177766
ZmNAC22	ZmNAC113	Si006829m	LOC_Os06g01480	Bradi1g52187	OG5_140455
ZmNAC23	ZmNAC52	Si007822m	LOC_Os06g33940	Bradi1g38730	OG5_160167
ZmNAC31	ZmNAC66	Si036757m	LOC_Os03g04070	Bradi1g76207	OG5_178238
ZmNAC35	ZmNAC86	Si001021m	LOC_Os01g15640	Bradi2g09530	NO_GROUP
ZmNAC41	ZmNAC79	Si030752m	LOC_Os07g12340	Bradi1g53770	OG5_135169
ZmNAC45	ZmNAC104	Si006639m	-	Bradi1g43840	OG5_213021
ZmNAC49	ZmNAC111	Si012319m	-	Bradi5g11247	OG5_212584
ZmNAC58	ZmNAC84	Si001527m	LOC_Os01g09550	-	OG5_190453
ZmNAC65	ZmNAC100	Si013361m,Si013378m,Si013524m	LOC_Os08g44820	Bradi3g12470	OG5_139552
ZmNAC69	ZmNAC72	Si022103m,Si022188m	LOC_Os05g34600	Bradi2g25150	OG5_164698

-represents no orthologous genes were found
