# Supplementary material for: Molecular Evolution and Expansion Analysis of the NAC Transcription Factor in Zea mays
Source: PLoS One. 2014 Nov 4;9(11):e111837. doi: 10.1371/journal.pone.0111837 (PMC4219692; doi:10.1371/journal.pone.0111837)
Supplement: Table S6 — EST frequencies of ZmNAC genes. (PDF) [file pone.0111837.s011.pdf]

Table S6. EST frequencies of ZmNAC genes.

ID	Number of EST	Number of root	Number of Immature ear	Number of leaf primordia	Tissue	Stage
ZmNAC7	2	2	0	0	root	3-4 days old
ZmNAC16	1	0	0	1	shoot	P7-P11 leaf
ZmNAC33	1	0	1	0	Immature ear	0.5 cm to 2 cm
ZmNAC35	1	1	0	0	root	3-4 days old
ZmNAC39	4	0	4	0	Immature ear	0.5 cm to 2 cm
ZmNAC54	9	6	0	3	root	3-4 days old
ZmNAC63	1	0	0	1	shoot	P7-P11 leaf
ZmNAC65	1	0	1	0	Immature ear	0.5 cm to 2 cm
ZmNAC96	1	0	1	0	Immature ear	0.5 cm to 2 cm
ZmNAC101	2	0	0	2	shoot	P7-P11 leaf
ZmNAC105	1	0	1	0	Immature ear	0.5 cm to 2 cm
ZmNAC109	4	0	4	0	Immature ear	0.5 cm to 2 cm
ZmNAC115	9	9	0	0	root	3-4 days old
ZmNAC124	1	0	1	0	Immature ear	0.5 cm to 2 cm
