# Supplementary material for: Molecular Evolution and Expansion Analysis of the NAC Transcription Factor in Zea mays
Source: PLoS One. 2014 Nov 4;9(11):e111837. doi: 10.1371/journal.pone.0111837 (PMC4219692; doi:10.1371/journal.pone.0111837)
Supplement: Table S7 — Selected some ZmNAC genes used for coexpression analysis in Genevestigator database. (PDF) [file pone.0111837.s012.pdf]

Table S7. Selected some ZmNAC genes used for coexpression analysis in Genevestigator database.

Protein	Locus name	Subfamily	Microarray probe
ZmNAC6	GRMZM2G003715	TIP	ZmAffx.785.1.A1_at
ZmNAC8	GRMZM2G008374	NAM	Zm.2445.1.A1_at
ZmNAC11	GRMZM2G014653	ATAF	Zm.4838.1.A1_at
ZmNAC12	GRMZM2G018436	NAM	Zm.4179.1.A1_at
ZmNAC13	GRMZM2G018553	ATAF	Zm.10172.1.A1_at
ZmNAC15	GRMZM2G027309	ONAC003	Zm.14731.1.S1_at
ZmNAC25	GRMZM2G043813	NAC1	ZmAffx.255.1.A1_at
ZmNAC28	GRMZM2G054252	SENU5	Zm.2131.2.A1_at
ZmNAC35	GRMZM2G064541	OsNAC8	Zm.15796.1.S1_at
ZmNAC36	GRMZM2G068973	ATAF	Zm.5882.1.A1_at
ZmNAC37	GRMZM2G069047	OsNAC7	Zm.18951.1.A1_at
ZmNAC41	GRMZM2G079632	ATAF	Zm.10189.2.A1_at
ZmNAC54	GRMZM2G104400	NAC2	Zm.6616.1.A1_at
ZmNAC59	GRMZM2G113950	NAC2	Zm.739.1.S1_at
ZmNAC64	GRMZM2G123667	ATAF	Zm.12113.1.A1_at
ZmNAC68	GRMZM2G134073	SENU5	Zm.14501.3.A1_at
ZmNAC74	GRMZM2G154182	NAM	Zm.5914.1.A1_at
ZmNAC79	GRMZM2G162739	ATAF	Zm.14805.1.A1_at
ZmNAC83	GRMZM2G163914	TIP	Zm.5270.1.S1_at
ZmNAC85	GRMZM2G167018	NAC1	Zm.7462.1.A1_at
ZmNAC89	GRMZM2G174070	ANAC011	Zm.7756.1.A1_at
ZmNAC90	GRMZM2G176677	NAC2	Zm.7735.1.S1_at
ZmNAC95	GRMZM2G181605	NAM	Zm.11843.1.A1_at
ZmNAC96	GRMZM2G312201	ATAF	Zm.10349.1.S1_at
ZmNAC102	GRMZM2G347043	ATAF	Zm.10147.1.A1_at
ZmNAC106	GRMZM2G389557	ONAC022	Zm.201.1.S1_at
ZmNAC107	GRMZM2G393433	NAM	Zm.15207.1.A1_at
ZmNAC109	GRMZM2G430522	NAM	Zm.4437.1.S1_at
ZmNAC115	GRMZM2G456568	NAC2	Zm.2490.1.A1_at
