# Supplementary material for: Molecular Evolution and Expansion Analysis of the NAC Transcription Factor in Zea mays
Source: PLoS One. 2014 Nov 4;9(11):e111837. doi: 10.1371/journal.pone.0111837 (PMC4219692; doi:10.1371/journal.pone.0111837)
Supplement: Table S8 — PCR primers used in this study. (PDF) [file pone.0111837.s013.pdf]

Table S8. PCR primers used in this study.

Gene name	Forward primer (5'-3')	Reverse primer (5'-3')
ZmNAC6	GCGCCGATTCCTTCAATGAC	ACACACAGTCTGGTTGCACA
ZmNAC13	CACCCCCACCCACCATC	CGGCTGTCCCATTATGTGGT
ZmNAC16	ACTCGGCTACAATGGCAACA	GCTTGTGCAAACCTTGGGAG
ZmNAC17	TCCAGTCCAAGCCCCCTTGC	CAAGCCTCTCCACGATCGGA
ZmNAC22	CTAGCTCACTCTTCTCGGCG	AAGATCAAAGGGAGGCCAGC
ZmNAC29	GCCGACCAGAGGAAGAAGAA	GTTCGGCCTGTTCTTCAGGA
ZmNAC31	CCACAGCAACATCCCTGGTC	GAGATGACGAGGACGGGAAC
ZmNAC34	TGCAGCCACAACTTCATTGC	GGGCACTTGCTCATAACCCT
ZmNAC35	CTGACGTGTGTCCGACTGAA	CTGGATAGGGCTAGCATGGC
ZmNAC41	GGAGAAGATGCAGCAGCAGA	GAAGTCCTCCACCTCCTCCT
ZmNAC45	CCTGGGGACAAGAATGCTCA	TTTTCCAGAGGGTGGGAGGA
ZmNAC50	ACCAGCAGCATCCATCGTTT	AGTATCTCTCGCCTTGGCTG
ZmNAC58	TGCTTGTTTGGTTTTGCTTTGC	CATGTCTGGTTCATACGCCG
ZmNAC63	ATGACACAAGCGGCAGGC	AGCTCGATGTTGAAACGCTTG
ZmNAC65	CAGCCAGATGAGCTGGAACA	TGGAATCGAACGGTGCATCA
ZmNAC66	AGCAGCTCCACTTCGACATC	ACTCCCTCTCCCCGATCTTC
ZmNAC67	CCTTCTTCCACCACCACCTC	GCTGCTGCTGGTTCAAGTTC
ZmNAC69	AATGCTACAAACGATGCCGC	TCCCAGCTAAGCTTGTCAGC
ZmNAC78	TGATAAACCAGGCGGAGGTG	CATGGCTCGCACTTGTTGAG
ZmNAC79	ACGCTCAGGTTGGATGACTG	CTTCTTCTCCTGCTGCTGCT
ZmNAC81	TCTACTACTGTGGCCGACGA	CGCAGGTACTCATCGAGGTC
ZmNAC83	TACAGATGGCACAGGTGCTG	CACCGTTGAACAAGTGCTGG
ZmNAC86	TCATCACCTCCAAACGGTGG	CTGGATAGGGCTAGCATGGC
ZmNAC100	ATGGAATTCTTGGCTCCGCA	GACGGGGCACCAACTAGAAA
ZmNAC102	CCGCGAGTGGTACTTCTTCA	CCTTCTTGATCCCGAGCGTG
ZmNAC104	TCAGGTTGTTCACCAGTGGC	ACACCTGGAAGTTTCTGTGGA
ZmNAC113	GGTGTGGATGCAATCTCAGGT	ATGGAACCGAAAGCCTGGAG
ZmHMG	GCTTGGTCTCCATGCTTCATCTAA	CGGTGAAACTGAACTGAACACAAC
